# Caprin-1 influences autophagy-induced tumor growth and immune modulation in pancreatic cancer

**DOI:** 10.1186/s12967-023-04693-4

**Published:** 2023-12-11

**Authors:** Wenbo Yang, Hongze Chen, Guanqun Li, Tao Zhang, Yuhang Sui, Liwei Liu, Jisheng Hu, Gang Wang, Hua Chen, Yongwei Wang, Xina Li, Hongtao Tan, Rui Kong, Bei Sun, Le Li

**Affiliations:** 1 Department of Pancreatic and Biliary Surgery, Key Laboratory of Hepatosplenic Surgery, Ministry of Education, The First Affiliated Hospital of Harbin Medical University, No.23 Youzheng St, Harbin, 150001 Heilongjiang China; 2 Department of General Surgery, Beijing Chaoyang Hospital of Capital Medical University, Beijing, China; 3 Department of Pharmacy, The First Affiliated Hospital of Harbin Medical University, Harbin, China; 4 Key Laboratory of Hepatosplenic Surgery, Ministry of Education, The First Affiliated Hospital of Harbin Medical University, Harbin, China

**Keywords:** Pancreatic ductal adenocarcinoma, Caprin-1, Autophagy, ULK1, STK38, Immune activation, CD4^+^T cells, Tumor-associated macrophage

## Abstract

**Background:**

Pancreatic ductal adenocarcinoma (PDAC) is characterized by rapid progression and poor prognosis. Understanding the genetic mechanisms that affect cancer properties and reprogram tumor immune microenvironment will develop new strategies to maximize the benefits for cancer therapies.

**Methods:**

Gene signatures and biological processes associated with advanced cancer and unfavorable outcome were profiled using bulk RNA sequencing and spatial transcriptome sequencing, Caprin-1 was identified as an oncogenesis to expedite pancreatic cancer growth by activating autophagy. The mechanism of Caprin-1 inducing autophagy activation was further explored in vitro and in vivo. In addition, higher level of Caprin-1 was found to manipulate immune responses and inflammatory-related pathways. The immune profiles associated with increased levels of Caprin-1 were identified in human PDAC samples. The roles of CD4^+^T cells, CD8^+^T cells and tumor associated macrophages (TAMs) on clinical outcomes prediction were investigated.

**Results:**

Caprin-1 was significantly upregulated in advanced PDAC and correlated with poor prognosis. Caprin-1 interacted with both ULK1 and STK38, and manipulated ULK1 phosphorylation which activated autophagy and exerted pro-tumorigenic phenotypes. Additionally, the infiltrated CD4^+^T cells and tumor associated macrophages (TAMs) were increased in Caprin-1^High^ tissues. The extensive CD4^+^T cells determined poor clinical outcome in Caprin-1^high^ patients, arguing that highly expressed Caprin-1 may assist cancer cells to escape from immune surveillance.

**Conclusions:**

Our findings establish causal links between the upregulated expression of Caprin-1 and autophagy activation, which may manipulate immune responses in PDAC development. Our study provides insights into considering Caprin-1 as potential therapeutic target for PDAC treatment.

**Supplementary Information:**

The online version contains supplementary material available at 10.1186/s12967-023-04693-4.

## Introduction

Pancreatic ductal adenocarcinoma (PDAC) is the fourth leading cause of death in malignant tumors. The treatments for PDAC are still challenging, due to delayed diagnosis and limited response to chemotherapy and immunotherapy [1, 2]. Many genomic targets have been discovered from human specimens in terms of advanced transcriptomic sequencing techniques, which provides more clue to improve early detection and strengthen target therapies in PDAC. Since half of PDAC is diagnosed at late stage and becomes unresectable, further studies are needed to understand the differences of tumor prosperities and gene signatures that can indicate tumor stages and the ability of metastasis.

PDAC has minimal T cells infiltration, while T cells are extensively enriched in some cases with pro-tumorigenic features rather than cytotoxic effects [3]. Due to the immunosuppressive dense fibrotic tumor microenvironment (TME) and tumor mutational burden, infiltrated cytotoxic T cells in PDAC are most likely to be functionally suppressed or exhausted [4]. Thus, understanding the characteristics that cause immune resistance and immune escape, as well as T cells mediated anti-tumor immunity will improve the response to immunotherapy.

Autophagy is indispensable for the modulation of tumor immunity [5, 6]. Therapy-induced immunogenic cell death elicits tumor immunogenicity and achieves tumor elimination. Autophagy can be activated by different therapies, accompanied with different types of infiltrated immune cells including CD8^+^T cells and myeloid-derived suppressor cells [7, 8]. Tumor-released autophagosomes are taken up by antigen presenting cells that drive anti-tumor adaptive immunity [9]. Our findings, consistent with previous studies, suggest that blockade of autophagy limits pancreatic cancer growth and overcomes chemotherapy and immunotherapy resistance [10, 11]. In view of the diversity and sensitivity of anti-tumor roles, targeting autophagy could be a promising pattern to sensitize PDAC to immunotherapy [12].

Here, we report that cytoplasmic activation/proliferation-associated protein-1 (Caprin-1) which is mainly involved in cell cycle regulation is upregulated in advanced and unresectable PDAC, as well as in short-term survival patients. Caprin-1 activates autophagy and overcomes cell death that can be implicated as a potential therapeutic target for PDAC. In addition, Caprin-1 recruits CD4^+^T cells and tumor associated macrophages (TAMs) infiltration in TME, indicating Caprin-1^High^ cancer cells are likely to escape from immune surveillance and fail to respond to immunotherapy. Our findings underline novel mechanisms of Caprin-1-induced autophagy activation and immunomodulation in PDAC progression, which provide evidence of targeting Caprin-1 on cancer treatment.

## Materials and methods

### Genes used for predicting the properties of PDAC

The top 100 differential expressed genes between early stage (pN = 0, pT ≤ 3) and advanced stage (pT = 4 or pM = 1) of PDAC were identified from GEO database (GSE62165). The different gene signatures between long-term survival and short-term survival were compared in GEO database (GSE84219) [13, 14]. The intersected gene signatures between tumor stage cohort (advanced *vs*. early stage) and survival cohort (short-term *vs*. long-term survival) were assessed. Kyoto Encyclopedia of Genes and Genomes (KEGG), Gene Ontology (GO) analysis and Ingenuity Pathway Analysis (IPA, Qiagen, Germany) were used to explore potential biological processes. The R package (ClusterProfiler) was used for data analysis.

### Mammalian cell lines

Pancreatic cancer cell lines Bxpc-3 and Panc-1 were obtained from American Type Culture Collection (ATCC, VT, USA). Human pancreatic duct epithelial (HPDE) cell line CFPAC and SW1990 were purchased from Cell Bank of the Chinese Academy of Sciences (Shanghai, China). Cells were cultured in 1640 (Gibco, MA, USA) or DMEM (Gibco) with 10% fetal bovine serum (FBS) and 1% penicillin–streptomycin (Thermo Fisher Scientific, MA, USA) at 37 °C with 5% CO_2_.

### Human specimen

All human experiments were approved by the ethics committee of the First Affiliated Hospital of Harbin Medical University (No.201822). Written informed consent for research use was obtained prior to specimen acquisition. One hundred and twenty-two PDAC samples were obtained from patients underwent radical resection from January, 2010 to January, 2018. All tissues were fixed and embedded in paraffin before conducting tissue microarray (TMA). In addition, seventy-four fresh PDAC tissues were stored in liquid nitrogen until use. Serum samples from forty-five healthy controls and sixty-nine pancreatic cancer patients were collected and stored at − 80 °C.

### Orthotopic engraftment and patient-derived xenograft (PDx) model

Six weeks female BALB/c nude mice and severe combined immunodeficient (SCID) mice were purchased from Vital River Lab Animal Technology Co., Ltd (Beijing, China). This animal study protocol was approved by the Institutional Review Board of the First Affiliated Hospital of Harbin Medical University. For orthotopic tumor model, 5 × 10^6^ Bxpc-3-Luc cells were injected into the left flank of BALB/c mice. Two weeks later, tumor was collected and cut into 2 × 2 mm^2^ pieces and transplanted into the tail of pancreas [10]. The peritoneum and abdominal wall were sutured with 6–0 absorbable vicryl sutures (Ethicon, NJ, USA). Mice were imaged weekly using in vivo imaging system (IVIS) (Berthold Technologies, Bad Wildbad, Germany). To develop patient-derived xenograft (PDx) mouse model, human PDAC tissues were collected from operation room and transfer to animal facility using DMEM medium. For the subcutaneous PDx model, the 2 × 2 mm PDAC tissues were transplanted in the left flack of SCID mice. The tumor size was measured weekly using caliper. For the orthotopic PDx model, tumor tissue was cut into 2 × 2 mm^2^ and was directly fixed on the tail of pancreas using 6–0 absorbable vicryl sutures. Mice were euthanized 9 weeks after tumor implantation.

### Spatial transcriptomics

Caprin-1^High^ (OS < 12 months) and Caprin-1^Low^ (OS > 60 months) formalin-fixed, paraffin-embedded (FFPE) PDAC tissues were stained by Hematoxylin and Eosin (H&E) staining, and bright-field images were acquired. A 10 μm FFPE tissue section were placed on Visium gene expression slide capture areas and permeabilization was performed using The Visium Spatial Tissue Optimization Slide & Reagent kit (10X Genomics, CA, USA). The sequencing libraries were conducted using Visium Spatial Gene Expression Slide & Reagent kit (10X Genomics), and sequencing was performed with a Novaseq PE150 platform (Illumina, CA, USA). For data processing, raw FASTQ files and histology images were processed by sample with the Space Ranger software (10X Genomics). The filtered gene-spots matrix and the fiducial-aligned low-resolution image was used for gene expression normalization, dimensionality reduction, spot clustering, and differential expression analysis using Seurat package. For spot clustering, the first 20 principal components (PCs) were used to build a graph, which was segmented with a resolution of 0.5. Wilcox algorithm was used to perform differential gene expression analysis for each cluster using FindAllMarkers function. Differential expressed genes with fold change > 2 and adjust *p value* < 0.05 were defined as significant.

### LC–MS/MS and data analysis

Proteins eluted from the Co-immunoprecipitation (Co-IP) assay were resolved by 10% SDS-PAGE gel. Protein bands were stained by the staining solution (0.1% Coomassie Blue R250 in 40% ethanol and 10% acetic acid) and destained. The in-gel digested samples were desalted using C18 ZipTip and loaded on a nano UPLC system (Waters, TX, USA) equipped with a self-packed C18 column. The peptides were eluted over 4 h into a nanoelectrospray ionization LTQ Orbitrap Velos mass spectrometer (Thermo Fisher Scientific). The Xcalibur^™^ software Version 4.1 (Thermo Fisher Scientific) was used to set the instrumental parameters and collect data. Raw data were searched against the UniProt human protein database containing 20,325 sequence entries and a common contaminants database using the Andromeda search engine embedded in the Mascot Software. Protein quantitation was performed in R using the unique peptide intensities exported from the Maxquant.

### Histopathological analysis and immunohistochemistry

Tissues were fixed in 10% neutral buffered formalin and embedded in paraffin. The 5 μm sections were subjected to H&E staining and immunohistochemistry (IHC). The primary antibodies were used for IHC were listed in Additional file 7: Table S3. According to the average number of positive cells (CD3, CD4, CD8 and CD68) or percentage of positive area (Caprin-1, ULK1 and STK38) in PDAC samples. Each marker was individually defined as high or low expressed based on the average expression level or number of positive cells across all samples.

### Statistical analysis

The clinical and pathologic parameters were analyzed with SPSS 26.0 software (IBM, NY, USA). The relevance between clinical parameters and the levels of Caprin-1 were analyzed by Chi-square test. The clinical features and OS were analyzed by Cox proportional-hazards model. The correlations between Caprin-1 and ULK1, STK38, CD4, CD68 and CD8 were assessed by *Pearson's* analysis. The statistical analyses of experiments were performed by GraphPad Prism 9.0. One-way ANOVA and *t*-test were used to evaluate statistical significance. The differences are considered significant when *p* < 0.05.

## Results

### High Caprin-1 level is associated with poor prognosis in PDAC

**Fig. 1:**
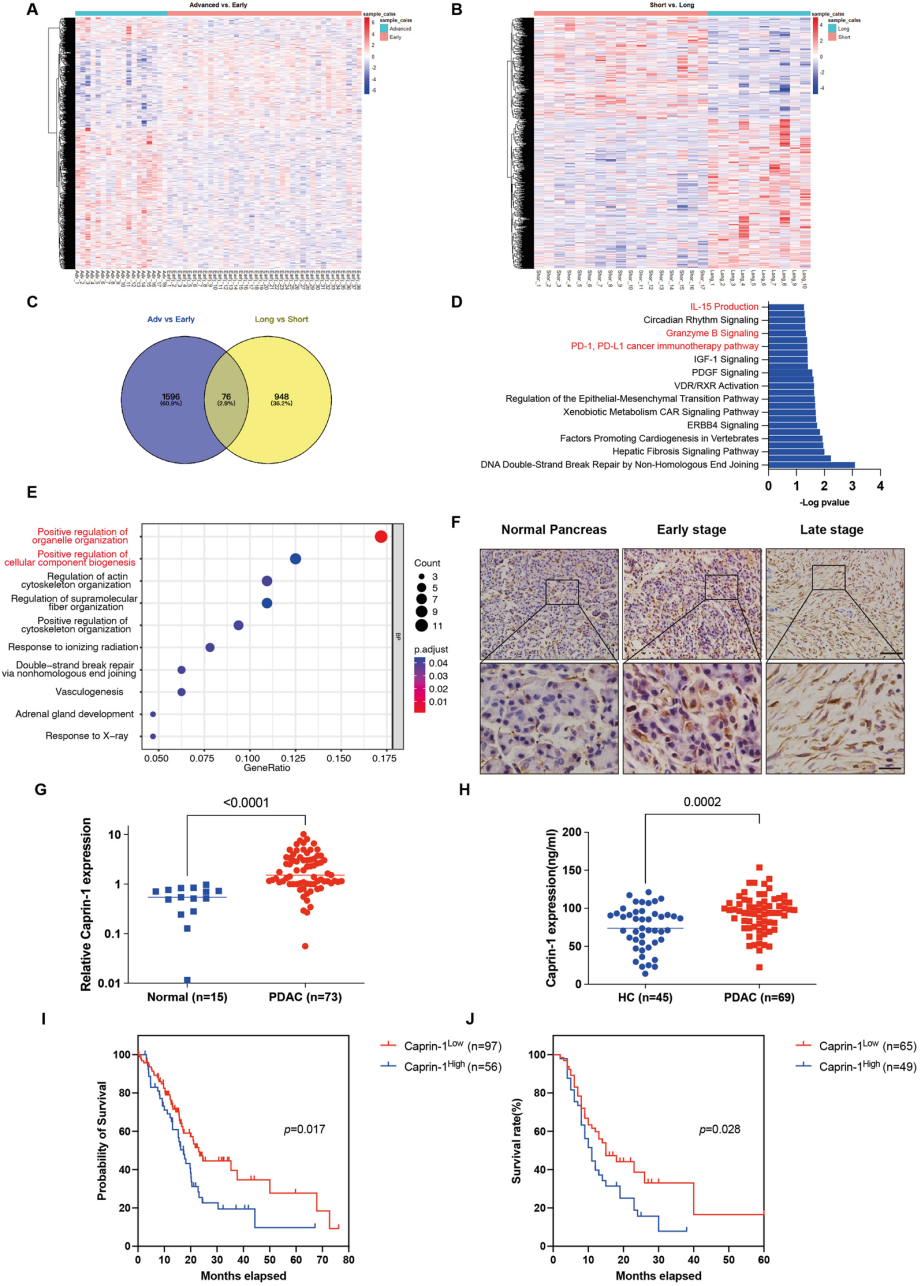
High level of Caprin-1 is associated with worse prognosis in PDAC. **A** Heatmap plotting the differential expressed genes between early stage and advanced stage in PDAC tissues. **B** Heatmap plotting differential expressed genes between short-term survival and long-term survival patients. **C** Venn diagram intersected gene signatures between advanced stage *vs.* early stage and short-term survival *vs.* long-term survival. **D** IPA analysis indicated significant profiles regulated by 76 different genes identified from two datasets. **E** GO analysis indicated top biological processes regulated by different genes. **F** IHC staining showed expression of Caprin-1 in human normal pancreas, early stage and advanced PDAC tissues. **G** The levels of Caprin-1 between adjacent and PDAC tissues were tested by qRT-PCR.** H** The serum levels of Caprin-1 between healthy volunteers and PDAC patients were examined by ELISA. **I** Kaplan–Meier curve showed the prognosis of PDAC patients between Caprin-1^High^ and Caprin-1^Low^ tissues from TCGA database. **J** The prognosis of PDAC patients between Caprin-1^High^ and Caprin-1^Low^ groups in our dataset

To investigate the genes that participate in PDAC development, RNA sequencing data from GEO database (GSE62165) was analyzed. PDAC tumors were separated at early stage (pN = 0, pT ≤ 3, pM = 0) and advanced stage (pT = 4 or pM = 1) using TNM stages classification. We found 1672 genes that were significantly changed in advanced stage compared to early stage (Fig. 1A). Furthermore, prognostic gene signatures between long-term survival and short-term survival were identified from GEO dataset (GSE84219), and 1,024 different genes were identified (Fig. 1B). Venn diagram showed 76 genes that were both shown in advanced stage tumors and short-term survival patients’ tumors (Fig. 1C, Additional file 8: Table S4). Next, the functional predictions of gene signatures between advanced stage *vs*. early stage were explored. In IPA analysis, the top five enriched pathways included IL-15 production, circadian rhythm, Granzyme B Signaling, PD-1, PD-L1 cancer immunotherapy pathway and IGF-1 signaling (Fig. 1D, Additional file 7: Table S5). GO analysis indicated that regulation of organelle organization, cellular component biogenesis and cytoskeleton organization were mainly enriched (Fig. 1E). As an oncogene, Carpin-1 was found to be dramatically increased in both gene sets, and has also been suggested to promote cancer growth in several previous studies [15–17]. We then tested the expression of Caprin-1 in normal pancreas and PDAC tissues, and found increased Caprin-1 expression in PDAC compared to normal pancreatic tissue, as well as that in advanced tumors compared to early stage tumors (Fig. 1F, G, Additional file 1: Fig. S1A, B). These indicate higher Caprin-1 level is positively associated with PDAC development. To explore whether Caprin-1 level could be used as a biomarker for tumor early detection, the serum levels of Caprin-1 in PDAC patients and healthy volunteers were compared, and increased level of Caprin-1 was found in PDAC patients compared with healthy volunteers (Fig. 1H). We then explored the role of Caprin-1 level on evaluating patients’ prognosis, the survival rates between Caprin-1^High^ and Caprin-1^Low^ PDAC from TCGA database were compared, and highly expressed Caprin-1 predicted poor clinical outcome (Fig. 1I). Furthermore, the associations between Caprin-1 expression in tumors and patients’ clinical parameters were investigated using two independent datasets, both of which suggested that higher Caprin-1 expression was associated with larger tumor size and poor prognosis (Fig. 1J, Additional file 7: Table S5, S6). Taken together, our results reveal that Caprin-1 promotes tumor progression and can be a biomarker for evaluating PDAC patients' prognosis.

### Spatial transcriptomics identify Caprin-1-associated heterogeneity in PDAC

**Fig. 2:**
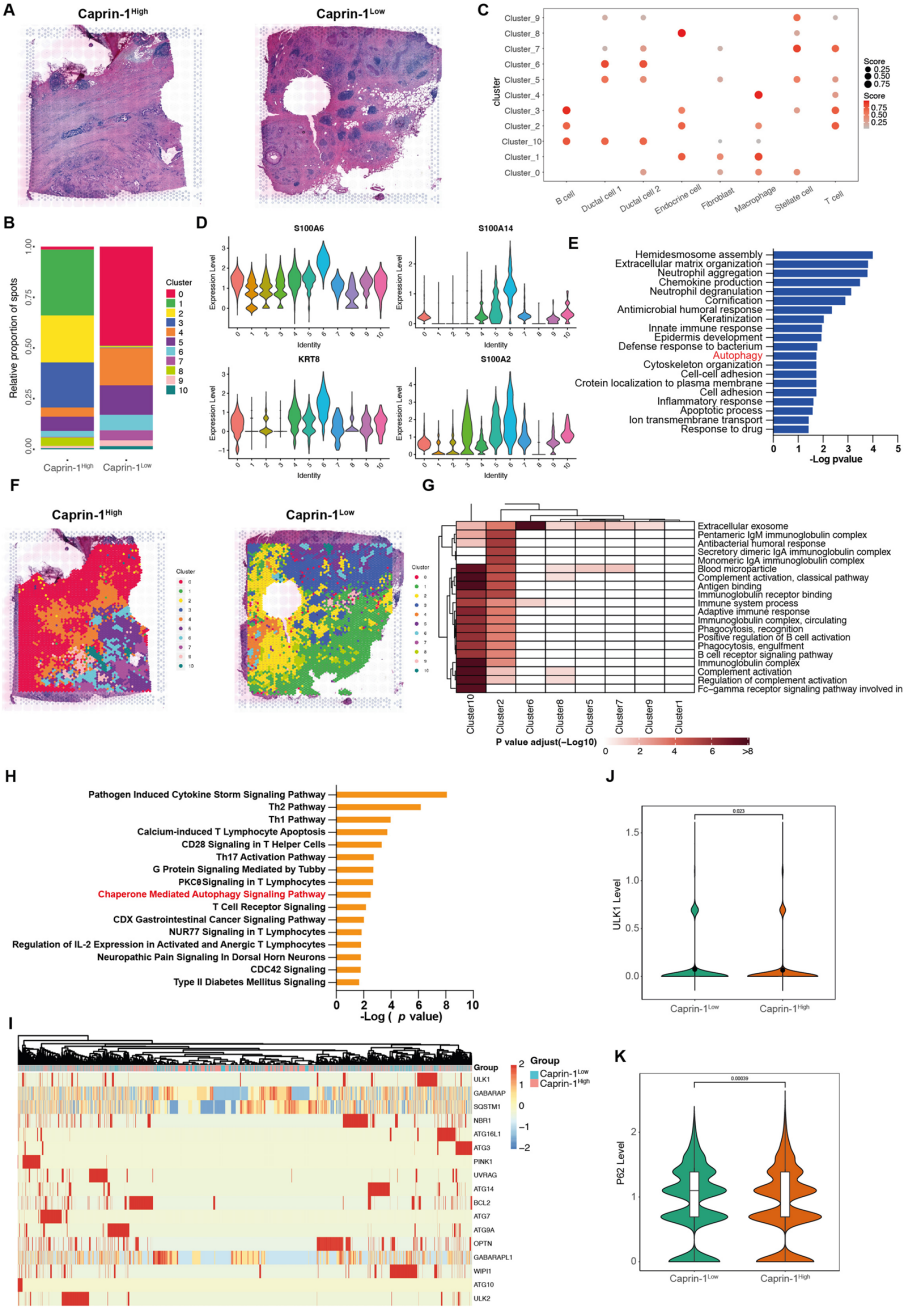
Spatial transcriptomics identify Caprin-1-associated heterogeneity in PDAC. **A** H&E staining for Caprin-1^High^ and Caprin-1 ^Low^ PDAC samples for spatial transcriptomic analysis. **B** The proportion of different clusters between Caprin-1^High^ and Caprin-1^Low^ tissues. **C** Dot plot showing the overlap between clusters and annotated regions. **D** Violin plot showing expressions of markers (S100A8, S100A14, KRT19 and S100A2) identified from ductal cells clusters. **E** Bar plot showing top 20 enriched biological process in ductal cells clusters using GO analysis. **F** Visualization of different clusters between Caprin-1^High^ and Caprin-1^Low^ tissues. **G** Heatmap showing enriched biological functions in all annotated clusters. **H** Bar plot showing top 15 pathways modulated by different genes in ductal cells between Caprin-1^High^ and Caprin-1^Low^ tissues. **I** Heatmap indicating the levels of 17 autophagy-related genes between Caprin-1^High^ and Caprin-1^Low^ tissues. **J**, **K** The levels of autophagy-related markers ULK and p62 between Caprin-1^High^ and Caprin-1^Low^ tissues

To elucidate the mechanisms of Caprin-1 influencing tumor development, Caprin-1^High^ tissue obtained from patients with poor prognosis (OS = 6 m) and Caprin-1^Low^ tumor with favorable outcome (OS = 60 m) were sequenced at spatial transcriptome levels (Fig. 2A). A total of 7961 captures and 17,323 genes were identified and integrated clustering analysis revealed 10 distinct clusters (Fig. 2B, C, Additional file 2: Fig. S2A). To understand the distribution of each cell types, clusters were mapped in terms of spatial locations and markers of each cluster were identified (Additional file 2: Fig. S2B). The expressions of markers identified from ductal cells were visualized and functional prediction suggested that autophagy was mainly activated in ductal cells compared to other cell types (Fig. 2D, E). Autophagy is an evolutionarily conserved process leading to the selective degradation of specific organelles in the vacuole/lysosomes. Autophagosomes engulf cytoplasmic material and organelles, including mitochondria, peroxisomes, lipid droplets, ribosomes. Our previous studies have also demonstrated that autophagy is crucial for PDAC development [10, 18, 19]. Thus, we hypothesized that Capirn-1 may affect tumor development through the regulation of autophagy. We then visualized the mainly enriched biological processes for each cluster and found immunoglobulin complex and immunomodulation, immunoglobulin receptor binding and adaptive immune response were enriched (Fig. 2F, G). The differential expressed genes in ductal cells were identified between Caprin-1^High^ and Caprin-1^Low^ tissues and chaperon mediated autophagy activation was found increased in Caprin-1^High^ sample using IPA analysis (Fig. 2H). We then visualized the levels of 15 well-known autophagy-related genes between Caprin-1^High^ and Caprin-1^Low^ tissues, and ULK1 and p62 were found significantly increased in Caprin-1^Low^ tissue (Fig. 2I–K). These suggest that higher Caprin-1 level modulates autophagy activation which affects PDAC development.

### Caprin-1 accelerates pancreatic cancer growth

**Fig. 3:**
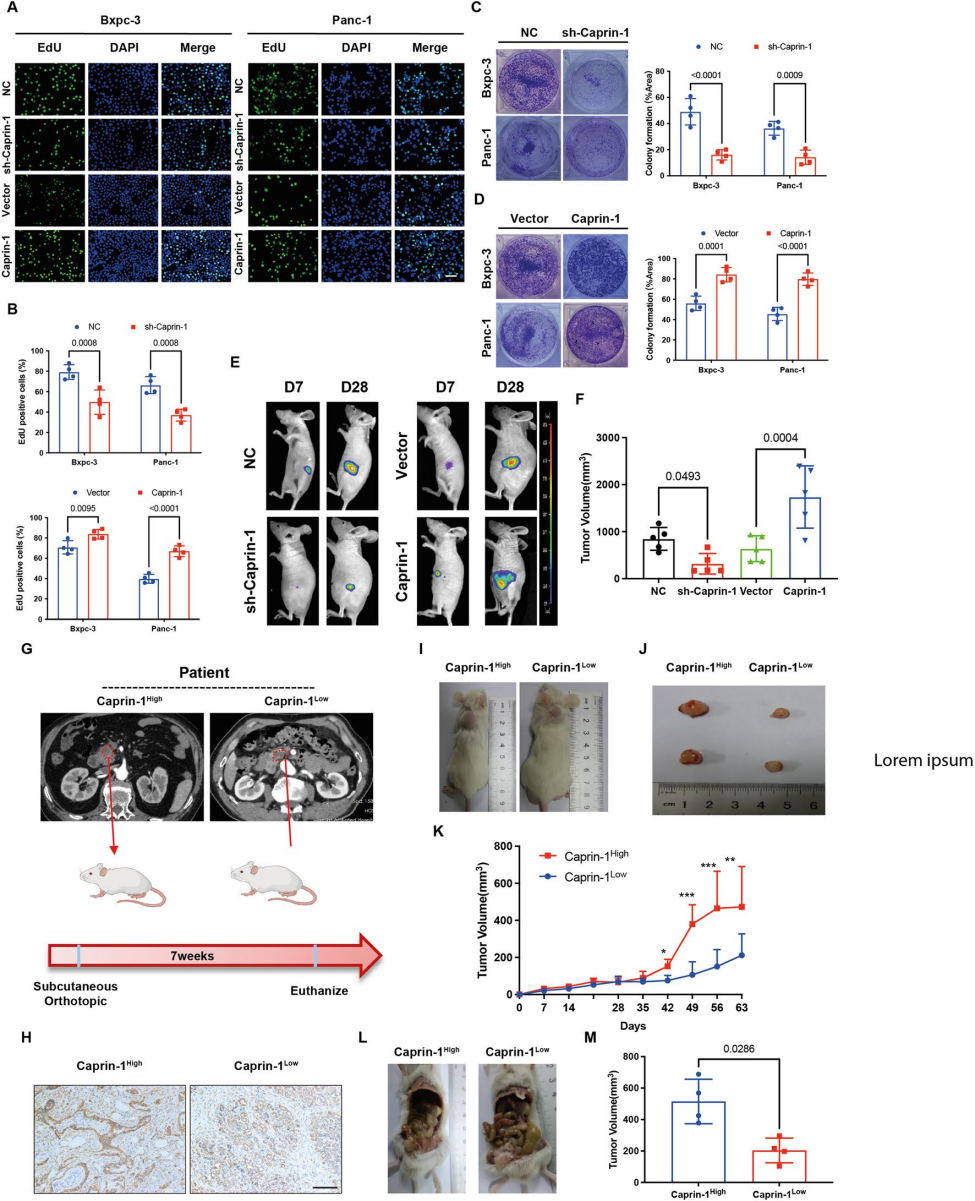
Caprin-1 promotes pancreatic cancer growth. **A**, **B** EdU assay verified the role of Caprin-1 on pancreatic cancer cells proliferation. **C**, **D** Colony formation showed the effects of sh-Caprin-1 and pcDNA-Caprin-1 on Bxpc-3 and Panc-1 cells growth. **E**, **F** The orthotopic tumor growth from day 7 to day 28 after tumor implantation was traced using IVUS. Mice were sacrificed at day 28 after tumor injection and tumor size was measured. **G** The schedule of building PDx model using two PDAC tissues from patients underwent pancreaticoduodenectomy. **H** Representative images of high and low expressions of Caprin-1 in PDAC samples by IHC staining. **I**–**K** The tumor size was compared between Caprin-1^High^ and Carpin-1^Low^ groups in subcutaneous PDx model. The tumor growth curve of subcutaneous PDx was screened from day 0 to day 63 after tumor injection. **L**, **M** The orthotopic PDx model was built and tumor volumes were compared between Caprin-1^High^ and Carpin-1^Low^ groups

To validate the traits of Caprin-1 on PDAC progression, the levels of Caprin-1 between pancreatic cancer cell lines and normal epithelium cell were compared. Caprin-1 was upregulated in most of pancreatic cancer cell lines except CFPAC (Additional file 3: Fig. S3A, B). We then investigated the roles of Caprin-1 on cancer cell proliferation using EdU cell proliferation assay and colony formation. We found that overexpression of Caprin-1 expedited tumor cells proliferation, and knockdown of Caprin-1 attenuated the phenotype (Fig. 3A–D, Additional file 3: Fig. S3C, D). Further study indicated that overexpression of Caprin-1 promoted tumor growth in vivo and knockdown of Caprin-1 limited tumor progression (Fig. 3E, F). We then built a PDx model to interrogate the pro-tumorigenesis role of Caprin-1. The Caprin-1^High^ and Caprin-1^Low^ human PDAC tissues were collected and transplanted into SCID mice (Fig. 3G, H). The significant difference of tumor size was found between Caprin-1^High^ and Caprin-1^Low^ groups from seven weeks after tumor implantation (Fig. 3I–K). The consistent results were also shown in orthotopic PDx mouse model (Fig. 3L, M). In addition, mice inoculated with Caprin-1^High^ tumor increased serum Caprin-1 levels (Additional file 3: Fig. S3E, F). These suggest Caprin-1 as a crucial regulator for tumor growth.

### Caprin-1 interacts with ULK1 and STK38 that activates autophagy and accelerates tumor growth

**Fig. 4:**
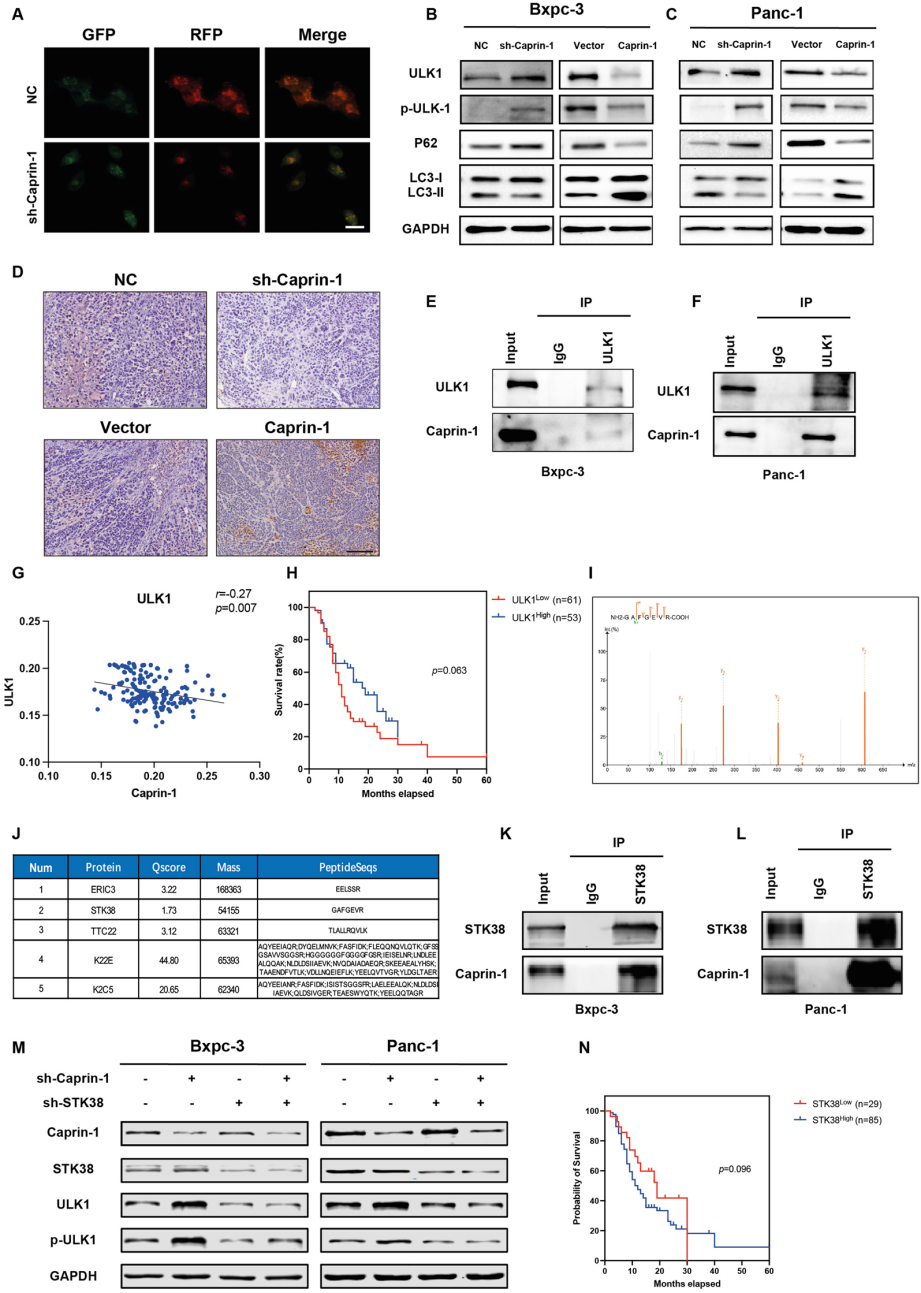
Caprin-1 interacts with ULK1 and STK38 that activates autophagy and drives tumor growth. **A** The autophagy flux was tracking by tandem fluorescent-tagged LC3 (mRFP-EGFP-LC3) assay in Panc-1 cells. **B**, **C** The expressions of autophagy genes LC3, p62, ULK1 and p-ULK1 were detected in cancer cells with knockdown or overexpression of Caprin-1. **D** The autophagy gene LC3B was stained in orthotopic tumors using IHC staining. **E**, **F** Co-IP assay validated the interaction between Caprin-1 and ULK1 in both Bxpc-3 and Panc-1 cell lines. **G** The level of ULK1 was tested in tumor microarray by IHC staining. **H** The Kaplan–Meier analysis compared the prognosis of patients with high and low expressed ULK1 in PDAC tissues. **I** The peptides interacted with Caprin-1 proteins were investigated by mass spectrometry and targeted proteins were predicted. **J** STK38 was predicted to bind with Caprin-1 in tumor cells. **K**, **L** The interaction between Caprin-1 and STK38 was certified by Co-IP assay.** M** The expressions of Caprin-1, STK38, ULK1 and p-ULK1 were examined in negative control, sh-Caprin-1, sh-STK38 and sh-Caprin-1 plus sh-STK38 groups in both Bxpc-3 and Panc-1 cells. **N** Kaplan–Meier curve plotted the overall survival of patients with high and low level of STK38 in PDAC

Since increased Caprin-1 is associated with autophagy activation, we then explore the regulatory effects of Caprin-1 on autophagy in PDAC. We found that knockdown of Caprin-1 decreased accumulation of autophagosomes in mRFP-GFP-LC3 assay, suggesting that deficiency of Caprin-1 blocked autophagy flux in pancreatic cancer cells (Fig. 4A). Autophagy influences pancreatic cancer proliferation and metastasis. LC3, a microtubule-associated protein, is subsequently localized to autophagosomes and isolation membranes during autophagy. LC3 is conjugated to phosphatidylethanolamine to form LC3-II, which is considered as an autophagosomal marker to reflect the number of autophagosomes [20]. Our results have shown that Caprin-1 level was negatively associated with Unc-51 like autophagy activating kinase 1 (ULK1) expression in PDAC. ULK1, also known as autophagic related gene 1 (ATG1), is an essential initiator for mammalian autophagy [21, 22]. The dephosphorylation of ULK1 induced autophagy activates pancreatic stellate cells (PSCs) and expedites pancreatic fibrotic process [23]. To further explore whether Caprin-1 accelerates tumor growth through modulating ULK1, the expression and phosphorylation levels of ULK1 were assessed between knockdown or overexpression of Caprin-1 in tumor cell lines. Our results revealed that Caprin-1 activated autophagy through impairing ULK1 expression and phosphorylation (Fig. 4B, C, Additional file 4: Fig. S4A–D). We then found that the intra-tumoral level of LC3B was attenuated using Caprin-1 knockdown Bxpc-3 cells (Fig. 4D).

ULK1 can interact with Caprin-1 and modulate stress granule disassembly [24]. We then verified the interaction between Caprin-1 and ULK1 and observed that Caprin-1 can directly interact with ULK1, we speculate that the interaction further affects ULK1 dephosphorylation (Fig. 4E, F). The interaction between Caprin-1 and p-ULK was also tested and we found that Capirn-1 failed to bind with p-ULK1 (Additional file 4: Fig S4E). Furthermore, we found a negative correlation between the levels of Caprin-1 and ULK1 in human PDAC tissues (Fig. 4G). Higher ULK1 score indicated better prognosis, which was consistent with the analysis data using TCGA database (Fig. 4H, Additional file 4: Fig. S4F). We also observed that knockdown of Caprin-1 increased ULK1 mRNA levels (Additional file 8: Fig. S5A–D). The mass spectrometry (MS) was performed to explore potential targets interacted with Caprin-1 (Additional file 5: Fig S5E, Additional file 4: Fig. S4I). A total of 278 peptides were identified and 105 proteins were ultimately predicted. The top five predicted proteins were listed, of which serine/threonine kinase 38 (STK38) was speculated as one potential downstream regulator of Caprin-1 (Fig. 4J). The kinase activity of STK38 is regulated by upstream kinases which induces its autophosphorylation or phosphorylation [25, 26]. The interaction between Caprin-1 and STK38 in pancreatic cancer cells were then confirmed (Fig. 4K, L). STK38 is implicated to suppress the transcription of ULK1 [26], we then explored the expressions of STK38, ULK1 and p-ULK1 in the absence of Caprin-1 or/and STK38 cancer cells. Our data conducted that downregulation of Caprin-1 had no effect on STK38 mRNA and proteins levels (Additional file 5: Fig. S5F, G). However, knockdown of Caprin-1 upregulated the expressions of ULK1 and p-ULK1, while the levels of ULK1 and p-ULK1 can be rescued by the inhibition of STK38 in Cparin-1 knockdown cells (Fig. 4M, Additional file 5: Fig S5H, I). Furthermore, we found that higher level of STK38 indicated poor prognosis in our dataset and TCGA database, suggesting that Caprin-1 promotes tumor growth through ULK1/STK38-dependent autophagy (Fig. 4N, Additional file 5: Fig. S5J).

### Caprin-1 induces T cells and macrophage infiltration in PDAC

**Fig. 5:**
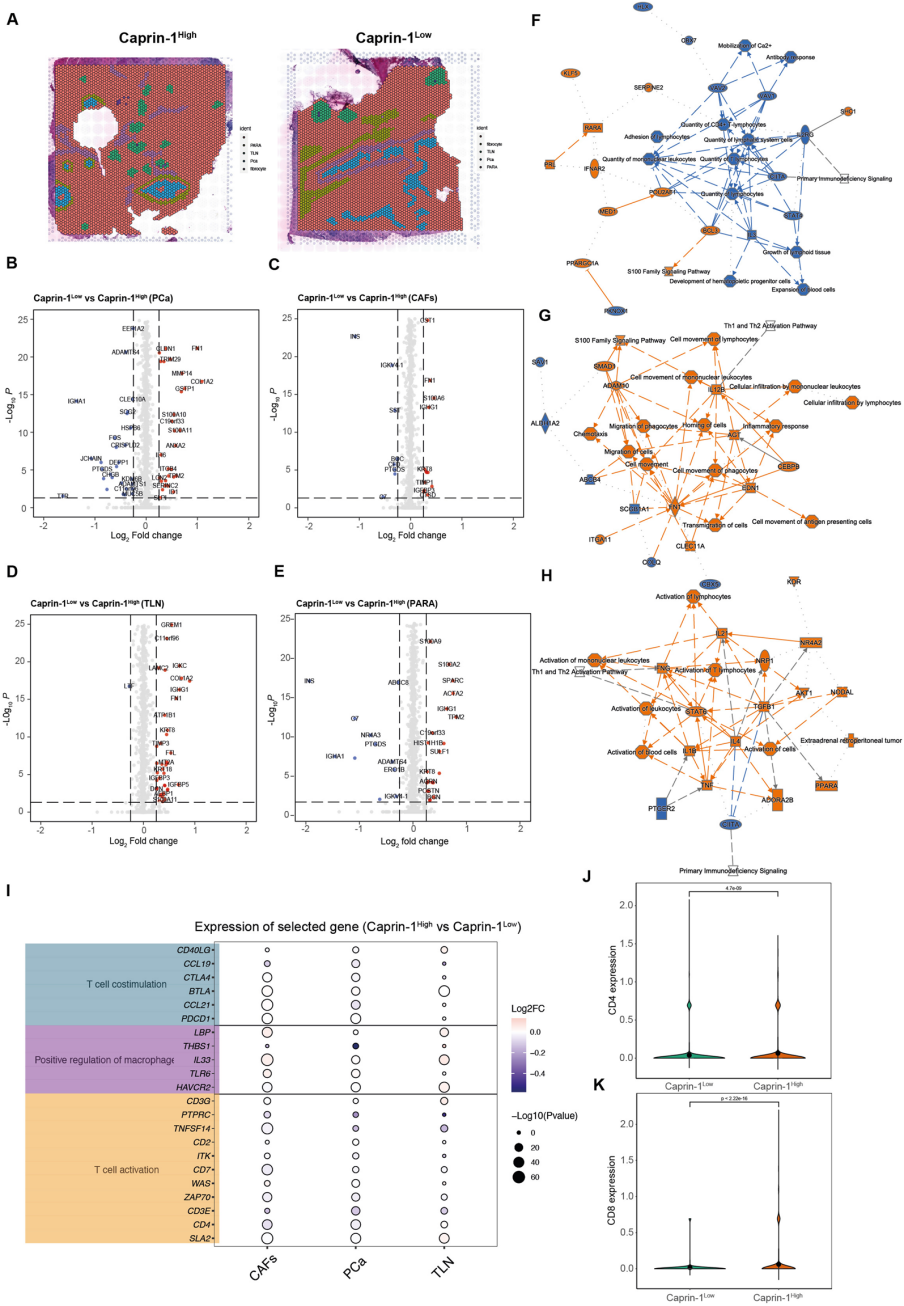
Caprin-1 induces T cells and macrophage infiltration in PDAC. **A** Visualization of tumor area (PCa), cancer-associated fibroblasts (CAFs), tertiary lymph nodes (TLN) and para-tumor area (PARA) in spatial level. **B** Volcano plot showing differential genes in PCa between Caprin-1^low^ and Carpin-1^high^ tissues. **C** Volcano plot showing differentially expressed genes in CAFs. **D** Volcano plot showing different genes in TNLs. **E** Volcano plot showing differentially expressed genes in TNLs. **F** The regulatory network generated by gene signatures identified from PCa. **G** The regulatory network generated by different genes found in CAFs. **H** The regulatory network generated by gene signatures identified from TLN. **I** The top enriched functions and gene signatures associated with T cell differentiation, positive regulation of macrophage and T cell activation in PCa, CAFs and TLN.** J**,** K** Relative levels of CD4 and CD8 between Carpin-1^high^ and Caprin-1^low^ tissues

To further approach differential biological processes between Caprin-1^High^ and Caprin-1^Low^ samples, cell types associated with pathology-annotated regions were selected (Fig. 5A). The differential genes in ductal cells (PCa), cancer-associated fibroblasts (CAFs), tertiary lymph nodes (TLN) and para-malignant ductal regions (PARA) were visualized (Fig. 5B–E). IPA analysis was applied to interpret the distinct cellular functions of each subpopulation. Caprin-1 was found to activate several immunological processes such as quantity of CD4^+^T lymphocytes, monocytes and T lymphocytes in ductal cells (Fig. 5F). In CAFs, Caprin-1 decreased the abilities of inflammatory response, leukocytes infiltration and antigen presenting cells movement (Fig. 5G). In TLN, Caprin-1 suppressed activation of lymphocytes, T cell activation and anti-tumor cytokine and chemokine (IFNG, IL1B, IL21 and TNF) production (Fig. 5H). These indicate that Caprin-1 may promote tumor growth through immune modulation and inflammatory-related pathways. We then explored regulatory effects by differential genes in PCa, CAFs and TLN using GO analysis and observed that T cell co-stimulation, positive regulation of macrophage and T cell activation were mainly enriched between Caprin-1^High^ and Caprin-1^Low^ tissues. The gene signatures enriched in these processes were visualized between PCa (CCL21, THBS1, PTPRC, TNFSF14, CD3E), CAFs (CCL19, THBS1, CD3E) and TLN (CCL19, CTLA4, PTPRC, TNFSF14, ITK, CD3E) (Fig. 5I). In addition, the expressions of CD4 and CD8 between Caprin-1^High^ and Caprin-1^Low^ tissues were compared, and Caprin-1 showed positive correlation with CD4^+^T cells infiltration, but was negatively correlated with CD8^+^T cell infiltration (Fig. 5J, K). These suggest that Caprin-1 potentiates immune surveillance and tumor progression by altering T cells and macrophage infiltration.

### ***Infiltration of CD4***^+^***T cells determine poor outcome in Caprin-1***^***High***^*** tumors***

**Fig. 6:**
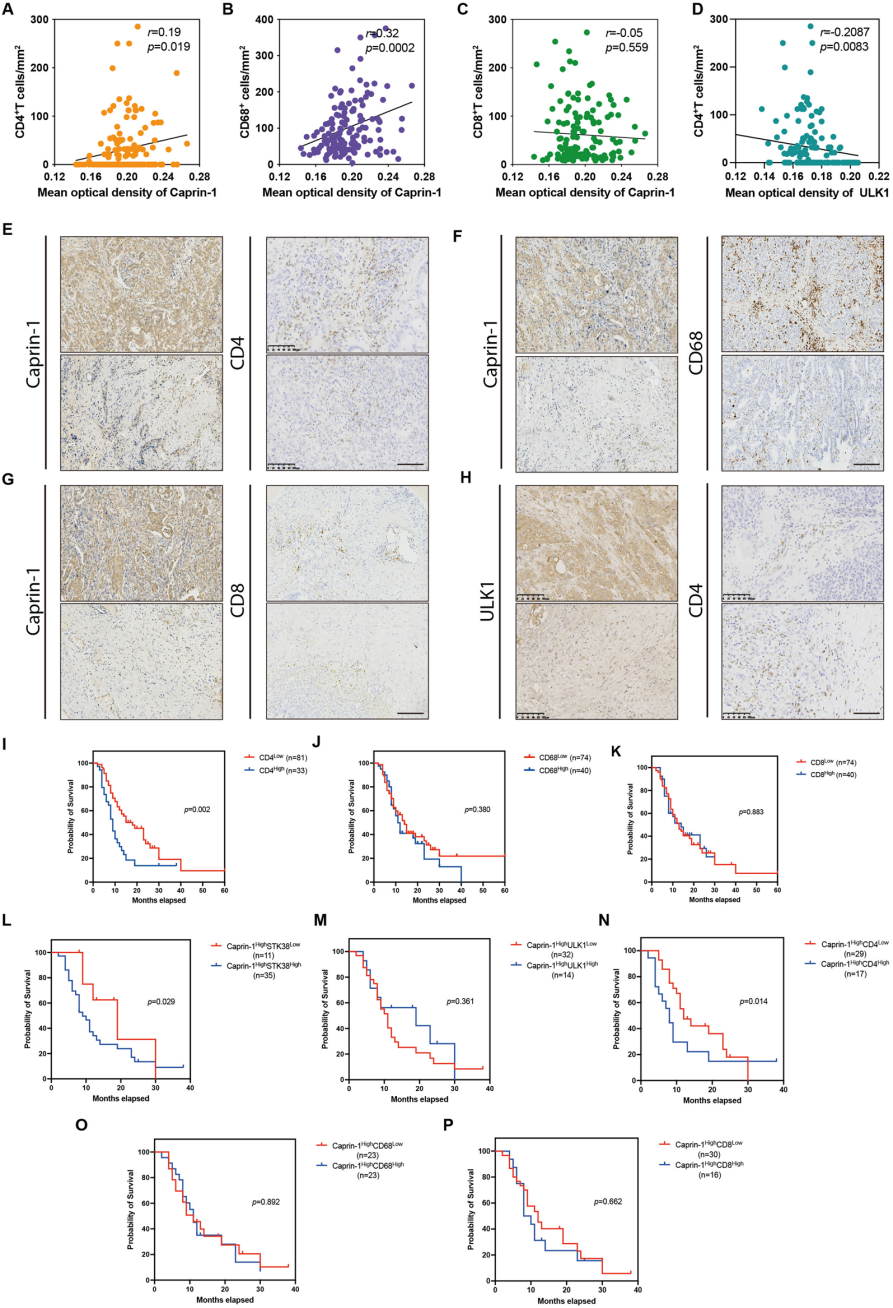
Infiltration of CD4^+^T cells determines poor outcome in Caprin-1^High^ tumors. **A**, **E** Correlation analysis between the expression of Caprin-1 and CD4^+^T cells infiltration in PDAC. **B**, **F** Correlation analysis between the expression of Caprin-1 and TAMs infiltration. **C**, **G** Correlation analysis between the level of Caprin-1 and infiltrated CD8^+^T cells. **D**, **H** Correlation analysis between the level of ULK1 and CD4^+^T cells infiltration. **I** Kaplan–Meier curves plotting survival curve of patients between high and low proportion of infiltrated CD4^+^T cells in PDAC. **J** Kaplan–Meier curve comparing the prognosis of patients between high and low proportion of infiltrated TAMs. **K** Kaplan–Meier curve plotting the survival of patients between high and low number of CD8^+^T cells infiltration. **L** Overall survival of patients according to high and low levels of STK38 in Caprin-1^High^ tissues. **M** Overall survival of patients between high and low levels of ULK1 in Caprin-1^High^ tumors. **N** Overall survival of patients according to high and low number of infiltrated CD4^+^T cells in Caprin-1^High^ tissues. **O** Kaplan–Meier curve plotted patients’ survival between high and low number of infiltrated TAMs in Caprin-1^High^ tissues. **P** Kaplan–Meier curve plotted the overall survival of patients between high and low proportion of infiltrated CD8^+^T cells in Caprin-1^High^ tissues

To further understand the roles of Caprin-1 on immune activation, the expressions of CD4^+^T cells, CD8^+^T cells and tumor-associated macrophages (TAMs) were quantified in PDAC tissues. The correlations between the expressions of Caprin-1 and ULK1, as well as numbers of infiltrated immune cells were assessed. Our results revealed that higher level of Caprin-1 was positively correlated with increased CD4^+^T cells and TAMs, but had no correlation with infiltrated CD8^+^T cells (Fig. 6A–C, E–G). In addition, ULK1 expression was negatively correlated with infiltrated CD4^+^T cells (Fig. 6D, H). We then investigated the roles of infiltrated CD4^+^T cells, TAM and CD8^+^T cells on predicting patient’s prognosis and found that higher population of CD4^+^T cells indicated poor OS, rather than TAMs and CD8^+^T cells (Fig. 6I–K). Next, patients were separated into Caprin-1^High^ and Caprin-1^Low^ subgroups according to average expressions of Carpin-1 in tumor, the effects of STK38 and ULK1 on evaluating OS were explored in Caprin-1^High^ patients. Higher level of STK38 indicated poor prognosis compared to lower group, while ULK1 level was inability of predicting patients’ outcome (Fig. 6L, M). We then assessed the roles of infiltrated CD4^+^T cells, TAMs and CD8^+^T cells on evaluating patients’ survival rates in Caprin-1^High^ subset and found that lower proportion of CD4^+^T cells was associated with better outcome, while TAMs and CD8^+^T cells showed no benefit on predicting patients’ prognosis (Fig. 6N–P). Our data demonstrate that Caprin-1-associated CD4^+^T cells and TAMs dominate immunosuppressive microenvironment that disclose poor prognosis in PDAC.

To explore the effect of Caprin-1 on immune modulation, autophagy activation in full immunocompetent mice, we firstly generated Caprin-1 knockdown Pan02 cell line, and orthotopically injected into the pancreas of mice (Additional file 6: Fig S6A, B). We found that knockdown of Caprin-1 slightly inhibited tumor growth, while the expression of LC3, as well as infiltrated numbers of CD4 and F4/80 positive cells were dramatically reduced. (Additional file 6: Fig S6C, D, E). We found no difference of CD8 positive cells between control and Caprin-1 knockdown tumors (Additional file 6: Fig S6D, E). In addition, the transcriptomic levels of ULK1 and STK38 were detected in tumor tissues and our results suggested that knockdown of Caprin-1 increased the expression of ULK1, while had no effect on STK38 level (Additional file 6: Fig. S6F).

## Discussion

**Fig. 7:**
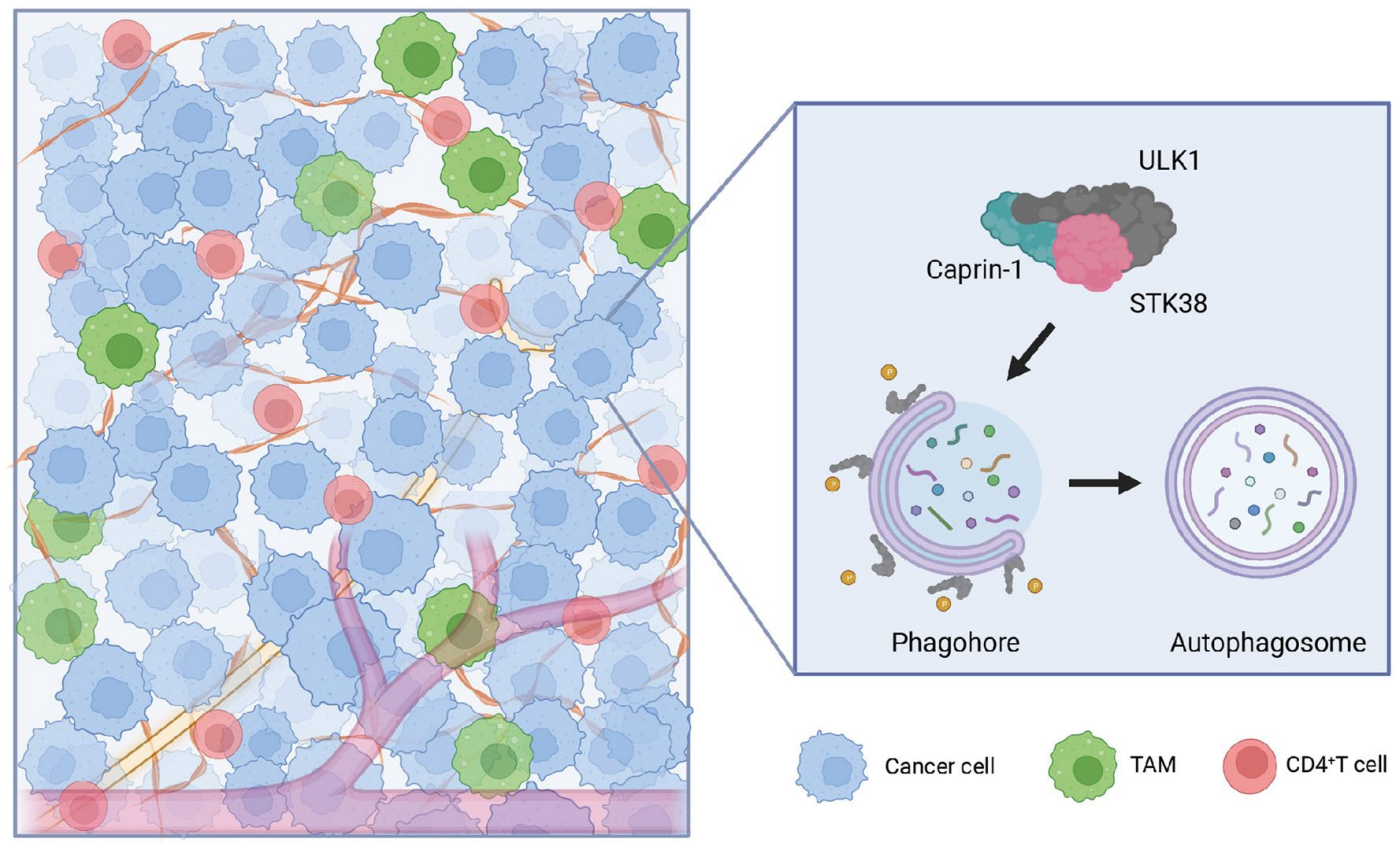
Graphical representation of the linkages between Caprin-1 dependent tumor-derived immunosuppression and autophagy in PDAC

Caprin-1 targets miRNAs or components of ribonucleoprotein complex and affects post-transcriptional modulations that are involved in pro-tumorigenic phenotypes [16, 27–29]. Caprin-1 has also been implicated in tumor-promoting inflammation, fibrosis and T cells recruitment [15, 30]. In current study, we profiled genes signatures in PDAC tissues between early stage *vs.* advanced stage and long-term survival patients *vs.* short-term survival patients. Caprin-1 was identified as an accelerator for PDAC development, and higher level of Caprin-1 indicated poor survival rate. Caprin-1 activated autophagy through the interaction with ULK1 and STK38 that promoted tumor growth. Simultaneously, Caprin-1 recruited CD4^+^T cells and TAMs that indicated poor outcome in Caprin-1^High^ PDAC patients. Our work reveals that Caprin-1-induced autophagy and immune activation influence PDAC progression and targeting Caprin-1 may play crucial roles against tumor development (Fig. 7).

Caprin-1 possesses RNA binding characteristics and affects cancer cells survival [27]. Most of work to date has focused on how Caprin-1 and its cooperated RNA-binding proteins drive downstream pathways that facilitate carcinogenesis [31]. Our study expands the knowledge of Caprin-1-dependent tumorigenesis through composing autophagy and the modulation of CD4^+^T cells and TAMs. Autophagy is a catabolic process in which cytoplasm bulk, proteins, and organelles are sequestered in autophagosomal vesicles and followed by lysosomal degradation [32, 33]. Our data demonstrates that Caprin-1 induces autophagy followed by an increase of the dephosphorylation of ULK1, a well-known molecular attributing to autophagosome formation. These findings align convincingly with our recent studies, suggest that autophagy is a key process enabling cancer cells viability and tumor metastasis. However, our prior work and current study demonstrate that autophagy promotes tumorigenesis through entirely different mechanisms. The "autophagy paradox" has been known as a pro-survival process and chemotherapeutics target by fueling cell metabolism [5, 34]. Our previous data suggests that elimination of autophagosome and lysosome fusion and autophagolysosome degradation attenuates PDAC growth [10]. Currently, we demonstrate that Caprin-1/ULK1/STK38 complex drives the initiation of autophagosome which contributes to tumorigenesis [35]. ULK1 has been shown to accelerate cancer cells growth [36], however, we found a positive correlation between higher level of ULK1 and extended prognosis in PDAC patients, suggesting ULK1 or autophagy associated heterogeneity in different cancer types. ULK1 plays pivotal roles in the initiation of autophagy [37, 38], our previous study uncovers that dephosphorylation of ULK1 induces autophagy and expedites pancreatic fibrosis [39]. Correspondingly, phosphorylation of ULK1 facilitates autophagosome fusion and links chaperone-mediated autophagy to macroautophagy [40]. Our data reveals that Caprin-1 induces the dephosphorylation of ULK1 and triggers autophagosome fusion that maintains cancer cells growth. STK38 is a Hippo pathway serine/threonine protein kinase with multifarious functions in cancers [41, 42]. STK38 supports the interaction of Exo84 with Beclin1 and RalB which is required for the initiation of autophagosome [42, 43]. Depletion of STK38 displays the impaired LC3B conversion and reduces ATG14L, ATG12, and WIPI-1 puncta formation [44]. Our findings demonstrate that Carpin-1 interacts with ULK1 and inhibits the phosphorylation of ULK1 that accelerates the initiation of autophagosome. Meanwhile, the interaction between Caprin-1 and STK38 diminishes ULK1 transcript and protein levels without impeding the expression of STK38.

PDAC patients with higher population of CD4^+^T cells and CD8^+^T cells exhibit favorable prognosis [45, 46]. In our study, higher proportion of CD8^+^T cells indicate no prolonged survival rate, suggesting that the infiltrated cytotoxic CD8^+^T cells in PDAC are possibly inactivated or exhausted. PDAC shows increased T regulatory cells infiltration which can suppress anti-tumor effectors including CD4^+^T cells and CD8^+^T cells. The inflammatory mediators or cytokines secreted by CD4^+^T cells can support immune evasion and maintain immune homeostasis that modulate anti-tumor immunity and pro-tumorigenic roles [47, 48]. We observe that higher number of CD4^+^T cells is relevant to shorter survival time in Caprin-1^High^ tumors. Further study needs to identify which subtypes of CD4^+^T cells are mainly recruited and the exact roles of remodeling immunosuppressive features. TAMs constitute to the predominant immune cells composition and are associated with poor prognosis in PDAC [49]. In terms of dynamic patterns, TAMs are commonly polarized to M2 deviation and contribute to tumor growth, cancer dissemination and drug resistance [50]. We found no correlation between infiltrated TAMs with patients’ outcomes. Besides, Caprin-1 recruits TAMs and blockade of Caprin-1 may hamper TAM-induced immunosuppressive effects and enhance anti-tumor response.

The consequences of autophagy on anti-tumor immunity have been described. Inhibition of autophagy can increase MHC-I level in tumor cells surface and trigger CD8^+^T cell infiltration, which might augment the response to immune checkpoint inhibitors (ICIs)^5^. TAMs, rather than T cells, upon inhibition of autophagy, also reinforce anti-tumor activity. Our findings hint the dual roles of autophagy in antigen presentation including the against of anti-tumor immune response and prevention of T cells-dependent tumor killing.

Here, we provide an avenue to elaborate PDAC treatment through the blockade of Caprin-1-indcued autophagy. Simultaneously, the positively correlations between Caprin-1 with infiltrated CD4^+^T cells and TAMs suggest that targeting Caprin-1 may overcome immune resistance and unleash response to ICIs.

### Supplementary Information

**Additional file 1: Fig S1.** The expressions of Caprin-1 in pancreatic cancer and normal epithelial cells. (A) The Caprin-1 protein levels were tested in normal pancreatic tissues and PDAC by Western blot. (B) The Caprin-1 expressions were tested in pancreatic adjacent tissues and PDAC using IHC staining (Scale bar=100μm).

**Additional file 2: Fig S2.** Spatial transcriptomics identify clusters and markers in PDAC samples. (A) tSNE embedding of spots colored by cluster identities. (B) Heatmap of clusters and top differentially expressed genes enriched in Carpin-1high and Caprin-1low tumors.

**Additional file 3: Fig S3.** Verification of knockdown and overexpression of Caprin-1 in tumor cell lines and relative Caprin-1 levels in PDx models. (A) The expressions and quantification of Caprin-1 and LC3 in pancreatic normal epithelial cell line and four pancreatic cancer cell lines. (B)The mRNA expressions of Caprin-1 in pancreatic normal epithelial cell line and tumor cell lines. (C) The efficacy of Caprin-1 knockdown in Panc-1 cells by qRT-PCR. (D) The efficacy of Caprin-1 overexpression in Panc-1 and Bxpc-3 cells by qRT-PCR. (E) Comparison of relative Caprin-1 levels between Caprin-1High and Caprin-1Low tumors in PDx model. (F) Comparison of relative Caprin-1 levels in the serum of Caprin-1High and Caprin-1Low PDx models.

**Additional file 4: Fig S4.** The associations between Caprin-1 and autophagy levels in cancer cells and the predictive roles of Caprin-1-associated genes in PDAC prognosis from TCGA database. (A, B) The quantification of ULK1, p-ULK1, P62 and LC3II/I in Caprin-1 knockdown and overexpression Bxpc-3 cells. (C, D) The quantification of ULK1, p-ULK1, P62 and LC3II/I in Caprin-1 knockdown and overexpression Panc-1 cells. (E) The interactions between Caprin-1 with p-ULK1, ULK1 and STK38 were detected by Co-IP assay. (F) Comparison of patients’ survival between high and low ULK1 expressed PDAC.

**Additional file 5: Fig S5.** The regulatory effects and interaction between Caprin-1 and ULK1, as well as STK38. (A, B) Relative Caprin-1 and ULK1 expressions in Caprin-1 knockdown and overexpression Bxpc-3 cells. (C, D) Relative Caprin-1 and ULK1 expressions in Caprin-1 knockdown and overexpression Panc-1 cells. (E) Identification of candidate proteins that bind with Caprin-1 using Coomassie Blue staining. (F, G) Relative STK38 expressions in Caprin-1 knockdown Bxpc-3 and Panc-1 cells. (H, I) The quantification of Caprin-1, STK38, ULK1 and p-ULK1 in the sh-Caprin-1, sh-STK38 or the combination of sh-Caprin-1 and sh-SKT38 groups. (J) Comparison of prognosis between high and low levels of STK38 in PDAC patients.

**Additional file 6: Fig S6.** The effects of Caprin-1 knockdown on tumor development in murine orthotopic tumor models. (A) Validation of Caprin-1 knockdown in Pan02 using qRT-PCR. (B) Validation of Caprin-1 knockdown in Pan02 by Western blot. (C) The comparison of tumor weight between NC and sh-Caprin-1 groups. (D, E) The expressions of Caprin-1, LC3, CD4, F4/80 and CD8 and their quantification in tumor tissues were compared between NC and sh-Caprin-1 groups. (F) The relative expressions of Caprin-1, ULK1 and STK38 in tumor tissues were compared between NC and sh-Caprin-1 groups.

**Additional file 7:** **Table S1.** Sequence of primers. **Table S2.** Antibodies for Western Blot. **Table S3.** Antibodies for IHC. **Table S5.** The association between Caprin-1 expression and clinical features in PDAC patients. **Table S6.** Survival analysis of variable features of PDAC patients.

**Additional file 8:** Intersection of gene signatures between early vs. late stage tumors and long-term vs. short survival patients.

## Data Availability

All data during the current study are available from the corresponding author upon reasonable request.

## Abbreviations

PDAC: Pancreatic ductal adenocarcinoma
Caprin-1: Cytoplasmic activation/proliferation-associated protein-1
TAMs: Tumor associated macrophages
TME: Tumor microenvironment
KEGG: Kyoto encyclopedia of genes and genomes
GO: Gene ontology
IPA: Ingenuity pathway analysis
TMA: Tissue microarray
PDx: Patient-derived xenograft
SCID: Severe combined immunodeficient
FFPE: Formalin-fixed, paraffin-embedded
IHC: Immunohistochemistry
PCa: Ductal cells
CAFs: Cancer-associated fibroblasts
TLN: Tertiary lymph nodes
ICIs: Immune checkpoint inhibitors
