# Supplementary figures and images for: Caprin-1 influences autophagy-induced tumor growth and immune modulation in pancreatic cancer
Source: J Transl Med. 2023 Dec 11;21:903. doi: 10.1186/s12967-023-04693-4 (PMC10714642; doi:10.1186/s12967-023-04693-4)

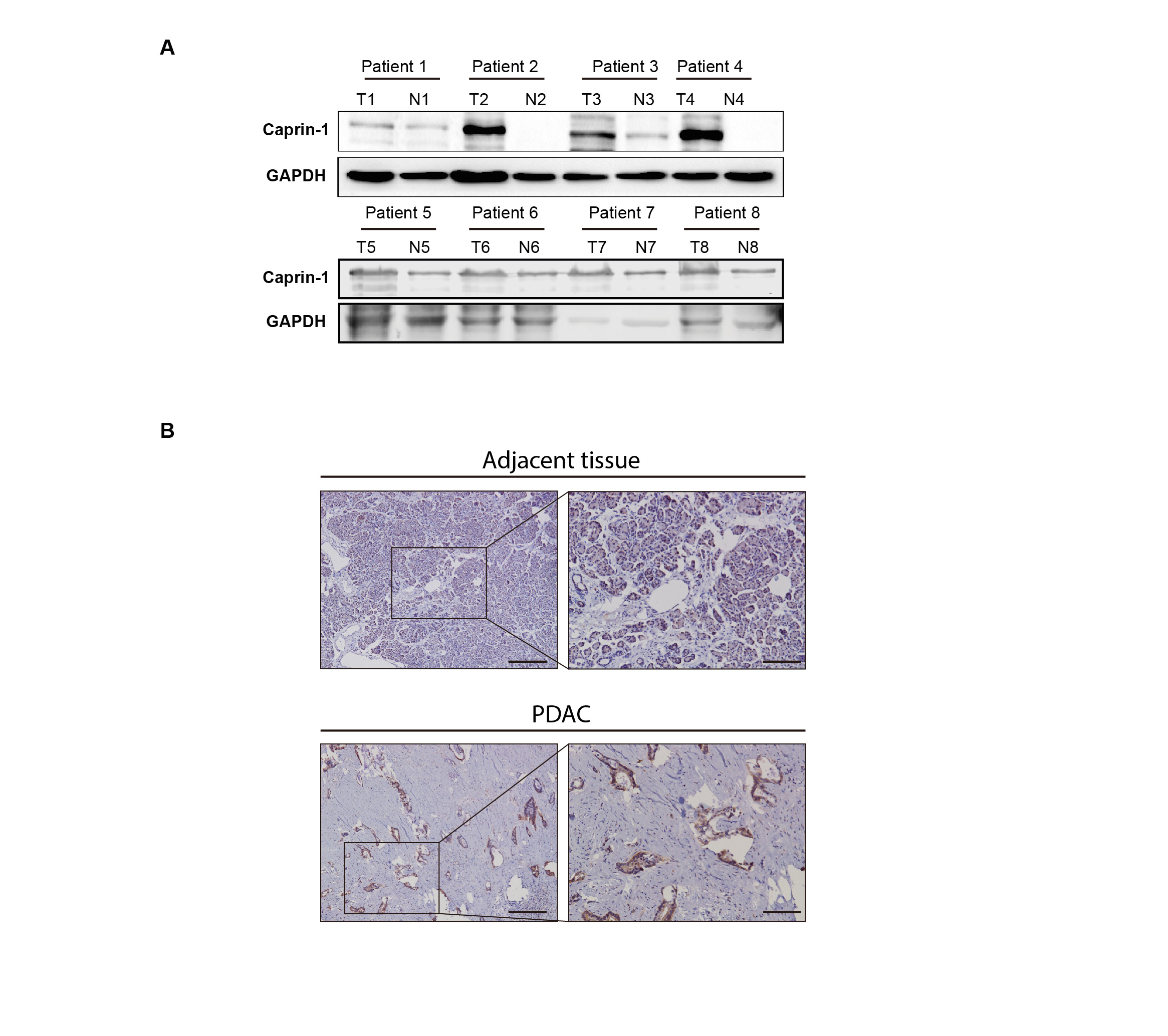

Supplement: Supplementary file 1 — Additional file 1: Fig S1. The expressions of Caprin-1 in pancreatic cancer and normal epithelial cells. (A) The Caprin-1 protein levels were tested in normal pancreatic tissues and PDAC by Western blot. (B) The Caprin-1 expressions were tested in pancreatic adjacent tissues and PDAC using IHC staining (Scale bar=100μm). [file 12967_2023_4693_MOESM1_ESM.jpg]

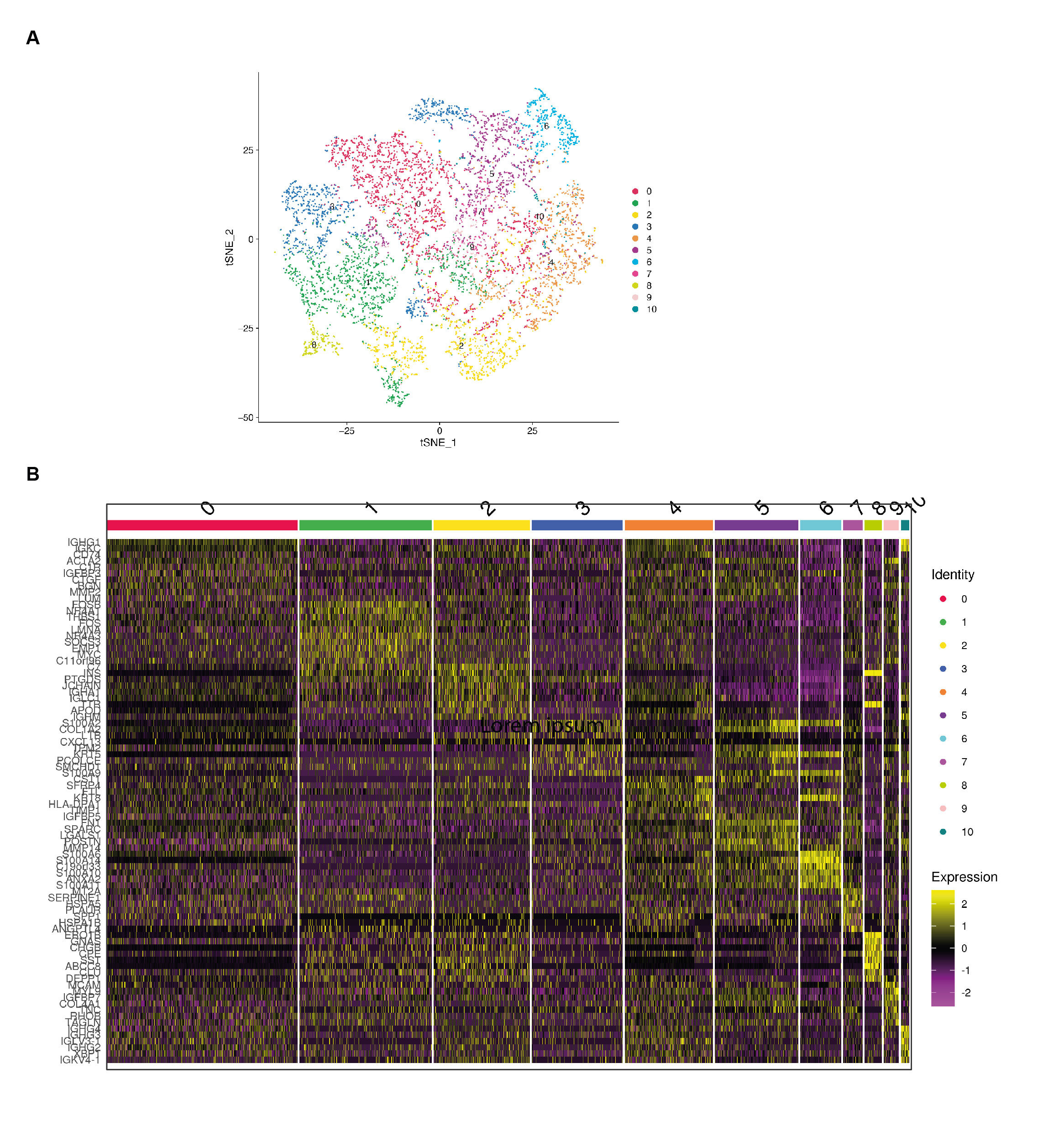

Supplement: Supplementary file 2 — Additional file 2: Fig S2. Spatial transcriptomics identify clusters and markers in PDAC samples. (A) tSNE embedding of spots colored by cluster identities. (B) Heatmap of clusters and top differentially expressed genes enriched in Carpin-1high and Caprin-1low tumors. [file 12967_2023_4693_MOESM2_ESM.jpg]

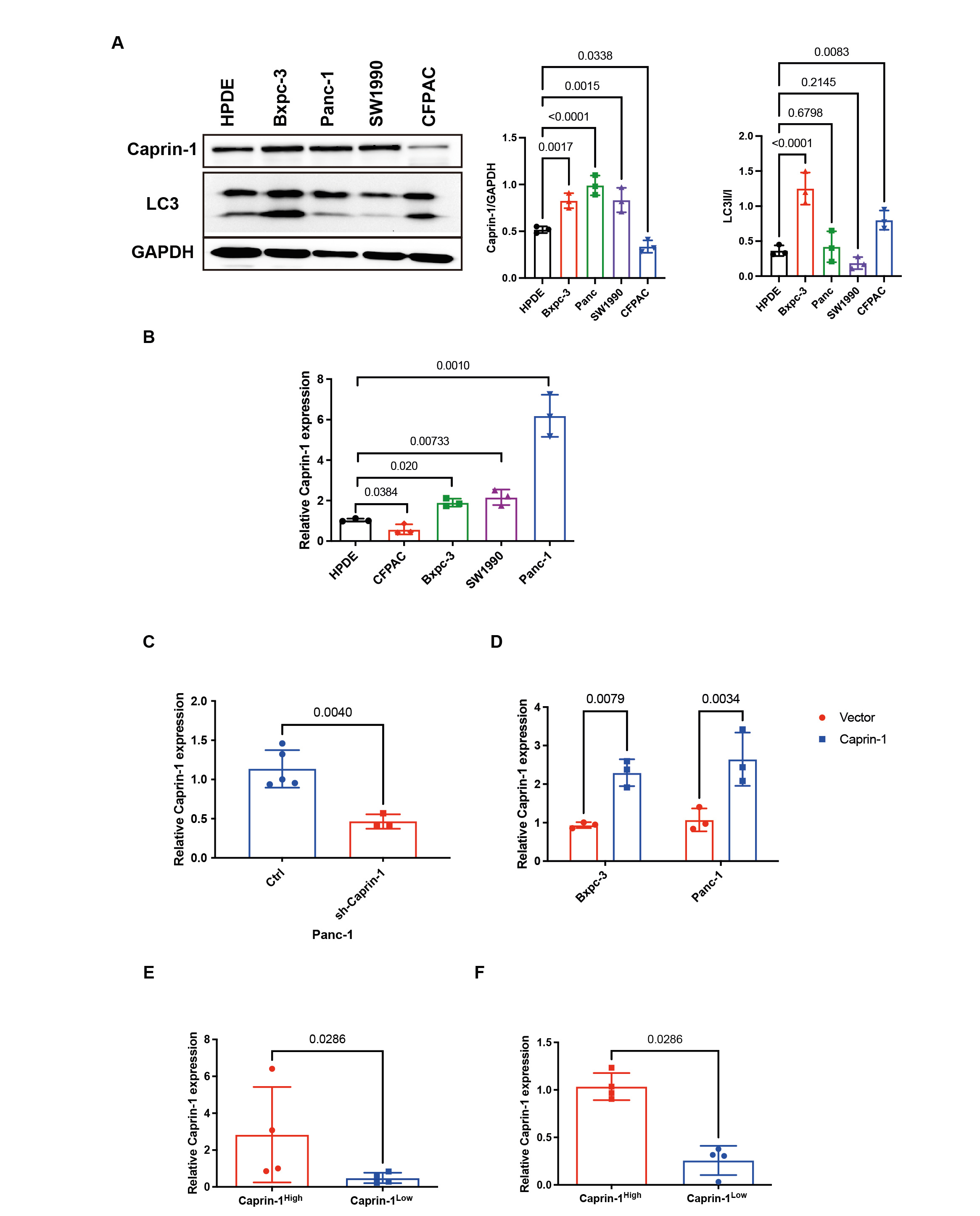

Supplement: Supplementary file 3 — Additional file 3: Fig S3. Verification of knockdown and overexpression of Caprin-1 in tumor cell lines and relative Caprin-1 levels in PDx models. (A) The expressions and quantification of Caprin-1 and LC3 in pancreatic normal epithelial cell line and four pancreatic cancer cell lines. (B)The mRNA expressions of Caprin-1 in pancreatic normal epithelial cell line and tumor cell lines. (C) The efficacy of Caprin-1 knockdown in Panc-1 cells by qRT-PCR. (D) The efficacy of Caprin-1 overexpression in Panc-1 and Bxpc-3 cells by qRT-PCR. (E) Comparison of relative Caprin-1 levels between Caprin-1High and Caprin-1Low tumors in PDx model. (F) Comparison of relative Caprin-1 levels in the serum of Caprin-1High and Caprin-1Low PDx models. [file 12967_2023_4693_MOESM3_ESM.jpg]

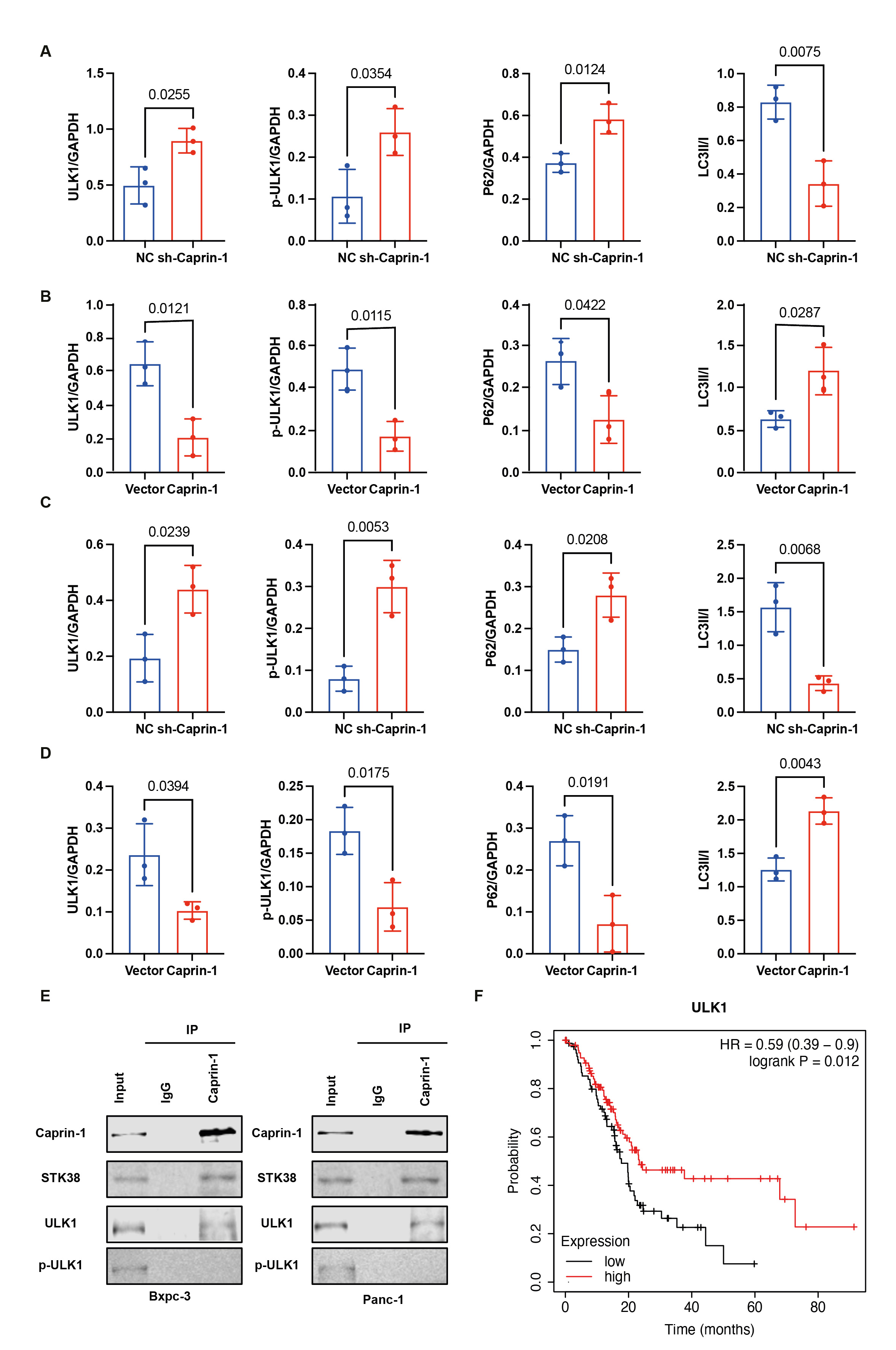

Supplement: Supplementary file 4 — Additional file 4: Fig S4. The associations between Caprin-1 and autophagy levels in cancer cells and the predictive roles of Caprin-1-associated genes in PDAC prognosis from TCGA database. (A, B) The quantification of ULK1, p-ULK1, P62 and LC3II/I in Caprin-1 knockdown and overexpression Bxpc-3 cells. (C, D) The quantification of ULK1, p-ULK1, P62 and LC3II/I in Caprin-1 knockdown and overexpression Panc-1 cells. (E) The interactions between Caprin-1 with p-ULK1, ULK1 and STK38 were detected by Co-IP assay. (F) Comparison of patients’ survival between high and low ULK1 expressed PDAC. [file 12967_2023_4693_MOESM4_ESM.jpg]

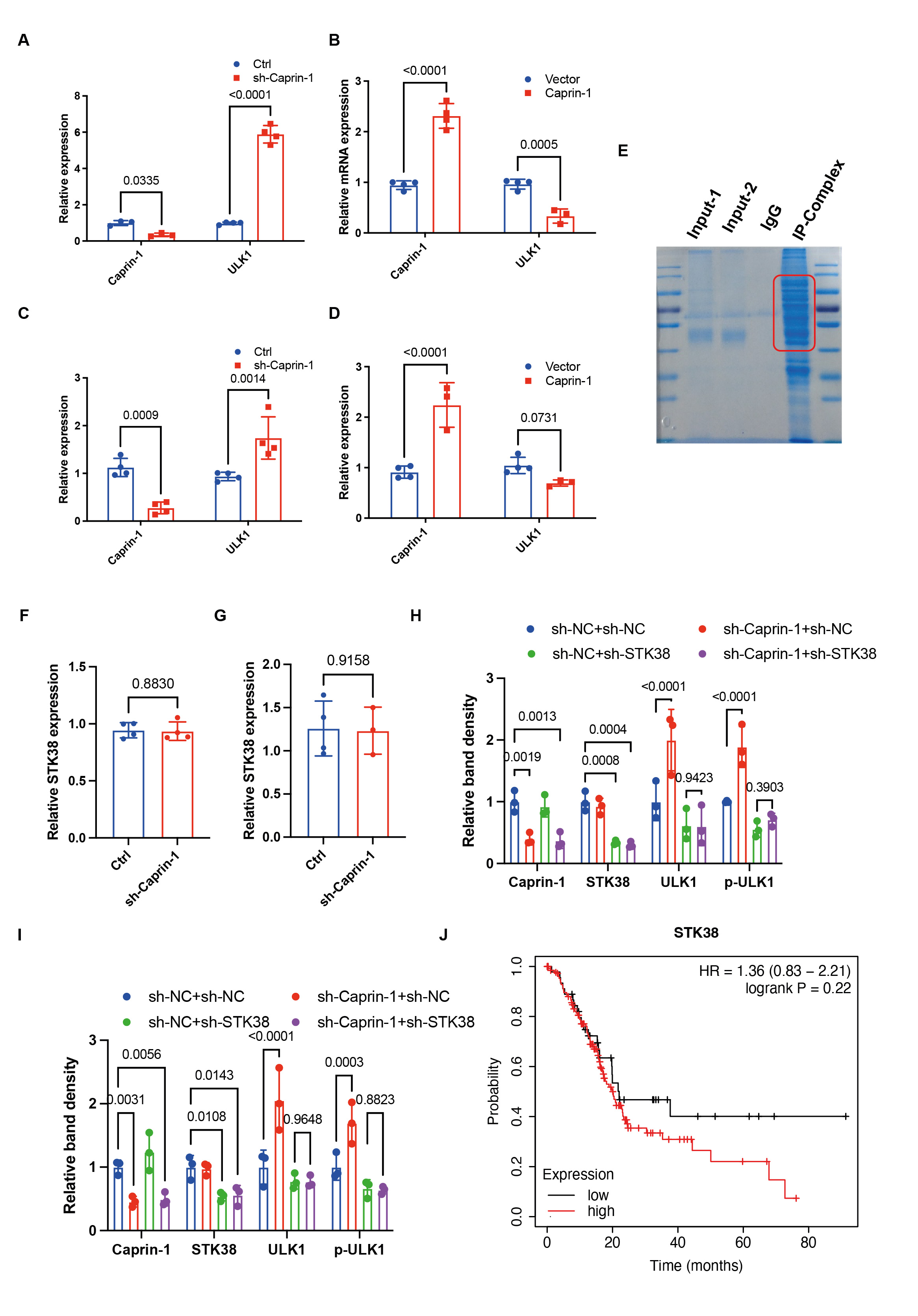

Supplement: Supplementary file 5 — Additional file 5: Fig S5. The regulatory effects and interaction between Caprin-1 and ULK1, as well as STK38. (A, B) Relative Caprin-1 and ULK1 expressions in Caprin-1 knockdown and overexpression Bxpc-3 cells. (C, D) Relative Caprin-1 and ULK1 expressions in Caprin-1 knockdown and overexpression Panc-1 cells. (E) Identification of candidate proteins that bind with Caprin-1 using Coomassie Blue staining. (F, G) Relative STK38 expressions in Caprin-1 knockdown Bxpc-3 and Panc-1 cells. (H, I) The quantification of Caprin-1, STK38, ULK1 and p-ULK1 in the sh-Caprin-1, sh-STK38 or the combination of sh-Caprin-1 and sh-SKT38 groups. (J) Comparison of prognosis between high and low levels of STK38 in PDAC patients. [file 12967_2023_4693_MOESM5_ESM.jpg]

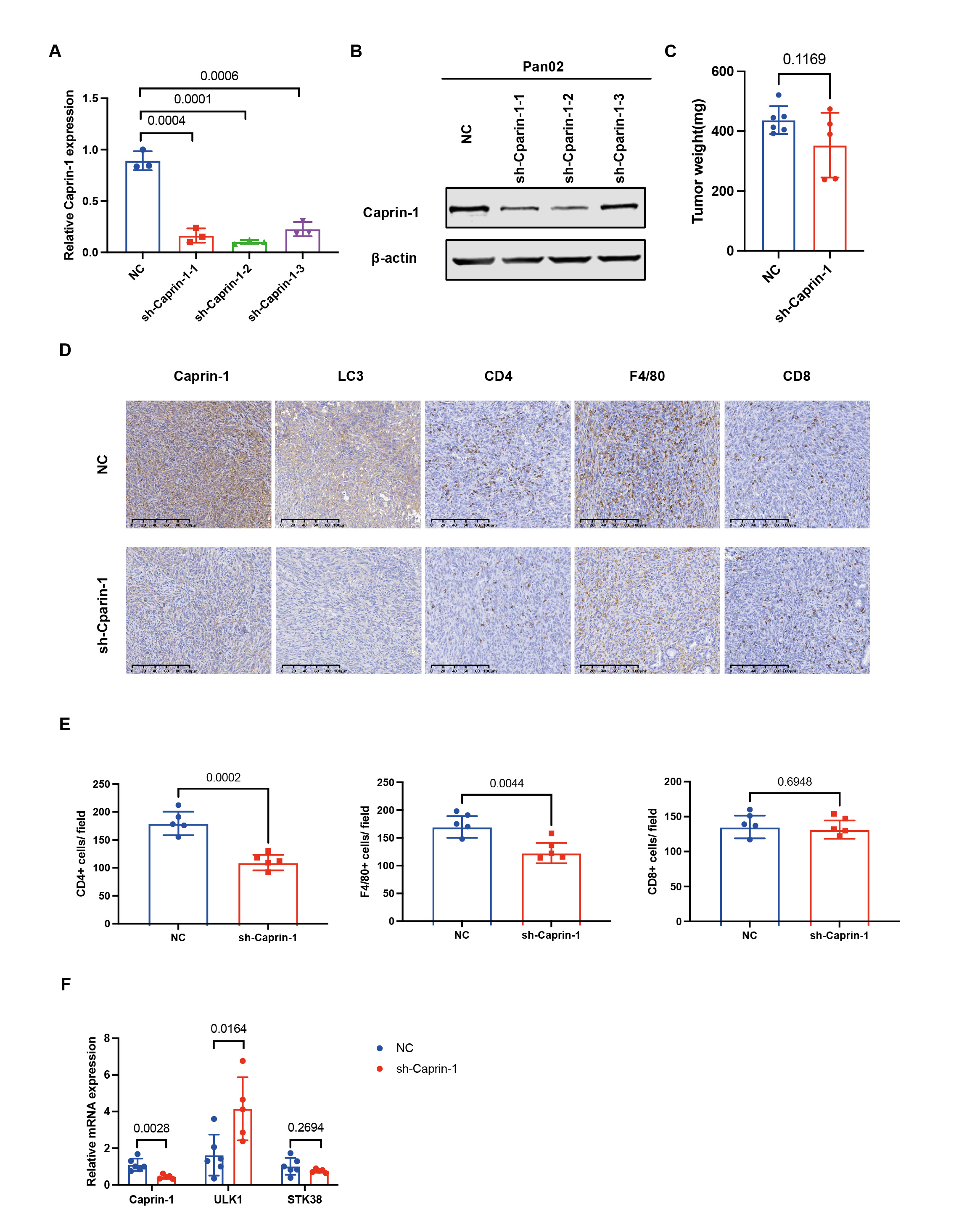

Supplement: Supplementary file 6 — Additional file 6: Fig S6. The effects of Caprin-1 knockdown on tumor development in murine orthotopic tumor models. (A) Validation of Caprin-1 knockdown in Pan02 using qRT-PCR. (B) Validation of Caprin-1 knockdown in Pan02 by Western blot. (C) The comparison of tumor weight between NC and sh-Caprin-1 groups. (D, E) The expressions of Caprin-1, LC3, CD4, F4/80 and CD8 and their quantification in tumor tissues were compared between NC and sh-Caprin-1 groups. (F) The relative expressions of Caprin-1, ULK1 and STK38 in tumor tissues were compared between NC and sh-Caprin-1 groups. [file 12967_2023_4693_MOESM6_ESM.jpg]
