# Supplementary material for: Caprin-1 influences autophagy-induced tumor growth and immune modulation in pancreatic cancer
Source: J Transl Med. 2023 Dec 11;21:903. doi: 10.1186/s12967-023-04693-4 (PMC10714642; doi:10.1186/s12967-023-04693-4)
Supplement: Supplementary file 7 — Additional file 7: Table S1. Sequence of primers. Table S2. Antibodies for Western Blot. Table S3. Antibodies for IHC. Table S5. The association between Caprin-1 expression and clinical features in PDAC patients. Table S6. Survival analysis of variable features of PDAC patients. [file 12967_2023_4693_MOESM7_ESM.docx]

**Additional file Material**

**1.Lentiviral infection**

The shRNA Caprin-1, pcDNA-Caprin-1 and mRFP-GFP-LC3 lentiviral vectors were purchased from Genechem (Shanghai, China). 1.0×10^6^ tumor cells were plated and infected with 5× concentrated virus with 5μg/ml polybrene. The infected cells were treated with 2μg/ml puromycin (Sigma-Aldrich, USA) for selection.

**2.Quantitative Real-time Polymerase Chain Reaction (qRT-PCR)**

RNA was extracted using the AxyPrep Multisource Total RNA Miniprep Kit (Axygen, CA, USA). cDNA was synthesized using ReverTra Ace™ qPCR RT Master Mix with gDNA remover Kit (Toyobo, Osaka, Japan). qRT-PCR was performed using SYBR Green protocol (Roche, Basel, Switzerland). The relative gene expression was quantified using 2^-ΔΔCT^ method and expression level was normalized to GAPDH. The primers were listed in the Supplementary Table 1.

**3.EdU retention assay**

2×10^4^ cells were seeded in 24-well plates and were stained with the Cell-Light EdU DNA Cell Proliferation Kit (RIBOBio, Guangdong, China). Cells were captured with confocal microscopy (Lecia, Germany).

**4.Colony formation assay**

Colony formation assay was performed as previously described. Five hundred cells were cultured in 6-well plates and medium was changed every three days. After 14 days, the colonies were counted with 1% crystal violet staining (Sigma-Aldrich, MO, USA). The percentage area of colony in 6-well plate was measured.

**5.Western Blot assay**

The cell lysates with 40μg of proteins were resolved on the SDS-PAGE and were subjected to Western blot assay. The primary antibodies were incubated overnight, followed by 1h incubation with secondary antibody. The bands were visualized using Molecular Imager System (Bio-Rad, CA, USA). The primary and secondary antibodies used were listed in the Supplementary Table 2.

**6.Co-IP assay**

1.0×10^6^ cells were lysed with 500µl of lysis buffer (100mM KCl, 5mM MgCl_2_, 10mM HEPES, 0.5% NP-40, 1mM dithiothrectol (Sigma-Aldrich), and Protease Inhibitor Cocktail (Roche) for 30min. Cell lysates were incubated with 5μg antibodies (anti-ULK1, ProteinTech, Hubei, China or anti-STK38, ProteinTech) to form protein-protein immunocomplexes, which were brought down by 20μl protein A/G agarose beads (Millipore, MA, USA). The complexes were collected and Western blot was performed.

**7.mRFP-GFP-LC3 assay**

The Panc-1 cells were transfected with GFP-mRFP-LC3 lentiviral vector (Genechem, Shanghai, China), followed by silencing or overexpressing Caprin-1. The autophagic vacuoles were visualized as RFP and GFP positive puncta. The number of fluorescent puncta was quantified in five randomized fields using confocal microscopy (Lecia, Wetzlar, Germany).

**8. ELISA assay**

The serum of PDAC patients were collected before surgery and stored at -80 °C for use. The concentration of Caprin-1 was determined by ELISA assay (CSB-PA614529LB01HU, Cusabio, China). Standards and 50 μL serum were both loaded in the same plate, and were incubated for 2 h at room temperature (RT). After removing unbound substances, the avidin-conjugated horseradish peroxidase (HRP) was added and incubated for 30 min at RT. Next, the stop solution was added and the optical density (OD) was obtained from a spectrophotometer using 450 nm as reference and 630 nm as correction wavelength (Thermo Fisher Scientific, USA). The standard curve was generated and the concentration was calculated.

**Figure Legend**

**Fig S1 The expressions of Caprin-1 in pancreatic cancer and normal epithelial cells.** (A) The Caprin-1 protein levels were tested in normal pancreatic tissues and PDAC by Western blot. (B) The Caprin-1 expressions were tested in pancreatic adjacent tissues and PDAC using IHC staining (Scale bar=100μm).

**Fig S2 Spatial transcriptomics identify clusters and markers in PDAC samples.** (A) tSNE embedding of spots colored by cluster identities. (B) Heatmap of clusters and top differentially expressed genes enriched in Carpin-1^high^ and Caprin-1^low^ tumors.

**Fig S3 Verification of knockdown and overexpression of Caprin-1 in tumor cell lines and relative Caprin-1 levels in PDx models.**

(A) The expressions and quantification of Caprin-1 and LC3 in pancreatic normal epithelial cell line and four pancreatic cancer cell lines. (B)The mRNA expressions of Caprin-1 in pancreatic normal epithelial cell line and tumor cell lines. (C) The efficacy of Caprin-1 knockdown in Panc-1 cells by qRT-PCR. (D) The efficacy of Caprin-1 overexpression in Panc-1 and Bxpc-3 cells by qRT-PCR. (E) Comparison of relative Caprin-1 levels between Caprin-1^High^ and Caprin-1^Low^ tumors in PDx model. (F) Comparison of relative Caprin-1 levels in the serum of Caprin-1^High^ and Caprin-1^Low^ PDx models.

**Fig S4 The associations between Caprin-1 and autophagy levels in cancer cells and the predictive roles of Caprin-1-associated genes in PDAC prognosis from TCGA database.** (A, B) The quantification of ULK1, p-ULK1, P62 and LC3II/I in Caprin-1 knockdown and overexpression Bxpc-3 cells. (C, D) The quantification of ULK1, p-ULK1, P62 and LC3II/I in Caprin-1 knockdown and overexpression Panc-1 cells. (E) The interactions between Caprin-1 with p-ULK1, ULK1 and STK38 were detected by Co-IP assay. (F) Comparison of patients’ survival between high and low ULK1 expressed PDAC.

**Fig S5 The regulatory effects and interaction between Caprin-1 and ULK1, as well as STK38.** (A, B) Relative Caprin-1 and ULK1 expressions in Caprin-1 knockdown and overexpression Bxpc-3 cells. (C, D) Relative Caprin-1 and ULK1 expressions in Caprin-1 knockdown and overexpression Panc-1 cells. (E) Identification of candidate proteins that bind with Caprin-1 using Coomassie Blue staining. (F, G) Relative STK38 expressions in Caprin-1 knockdown Bxpc-3 and Panc-1 cells. (H, I) The quantification of Caprin-1, STK38, ULK1 and p-ULK1 in the sh-Caprin-1, sh-STK38 or the combination of sh-Caprin-1 and sh-SKT38 groups. (J) Comparison of prognosis between high and low levels of STK38 in PDAC patients.

**Fig S6 The effects of Caprin-1 knockdown on tumor development in murine orthotopic tumor models.** (A) Validation of Caprin-1 knockdown in Pan02 using qRT-PCR. (B) Validation of Caprin-1 knockdown in Pan02 by Western blot. (C) The comparison of tumor weight between NC and sh-Caprin-1 groups. (D, E) The expressions of Caprin-1, LC3, CD4, F4/80 and CD8 and their quantification in tumor tissues were compared between NC and sh-Caprin-1 groups. (F) The relative expressions of Caprin-1, ULK1 and STK38 in tumor tissues were compared between NC and sh-Caprin-1 groups.

**Additional file Tables**

**Table S1 Sequence of primers**

| **Gene** | **Forward** | **Reverse** |
| --- | --- | --- |
| **Human** |  |  |
| Caprin-1 | ACCAGTGGTGAAAAGGAGCAGG | TGCCTGAGCCACTGGAGTCAAA |
| ULK1 | GCAAGGACTCTTCCTGTGACAC | CCACTGCACATCAGGCTGTCTG |
| STK38 | TCCACAGTAGGCACTCCTGACT | GGGTCTCAGAACAGAAAGGTGG |
| GAPDH | GTCTCCTCTGACTTCAACAGCG | ACCACCCTGTTGCTGTAGCCAA |
| **Mouse** |  |  |
| Caprin-1 | GTCAGATCCACTTGTGAGAAGGC | CTGTGCGGATACAATGGCAGGA |
| ULK1 | GCAGCAAAGACTCCTGTGACAC | CCACTACACAGCAGGCTATCAG |
| STK38 | ACAAGCTCTGCGATTGGTGGTC | TCTCGGAGACAGGAACTTCTGG |
| GAPDH | CATCACTGCCACCCAGAAGACTG | ATGCCAGTGAGCTTCCCGTTCAG |

**Table S2 Antibodies for Western Blot**

| **Antibody** | **Brand** | **Cat number** | **Concentration** |
| --- | --- | --- | --- |
| Caprin-1 | Proteintech | 15112-1-AP | 1:1000 |
| ULK1 | Cell Signaling Technology | 8054 | 1:1000 |
| p-ULK1(Ser555) | Cell Signaling Technology | 5869 | 1:1000 |
| LC3 | Cell Signaling Technology | 12741 | 1:1000 |
| P62 | Cell Signaling Technology | 39749 | 1:1000 |
| STK38 | Proteintech | 55335-1-AP | 1:1000 |
| STK38 | Santa Cruz | sc-271703 | 1:100 |
| GAPDH | Kangcheng Biotechnology | KC-5G5 | 1:3000 |

**Table S3 Antibodies for IHC**

| **Antibody** | **Brand** | **Cat number** | **Concentration** |
| --- | --- | --- | --- |
| Caprin-1 | ProteinTech | 15112-1-AP | 1:500 |
| ULK1 | Cell Signaling Technology | 8054 | 1:200 |
| LC3 | Cell Signaling Technology | 12741 | 1:100 |
| CD4 | Cell Signaling Technology | 48274 | 1:200 |
| CD68 | Cell Signaling Technology | 26042 | 1:200 |
| CD3 | Cell Signaling Technology | 85061 | 1:200 |
| CD8 | Cell Signaling Technology | 85336 | 1:200 |

**Table 5 The association between Caprin-1 expression and clinical features in PDAC patients**

| **Variable** | **N=76** | **Caprin-1** | | **x^2^** | **P Value** |
| --- | --- | --- | --- | --- | --- |
|  |  | **Low (N=52)** | **High (N=24)** |  |  |
| Age |  |  |  | 1.63 | 0.202 |
| ≤60y | 49 | 36 | 13 |  |  |
| >60y | 27 | 16 | 11 |  |  |
| Gender |  |  |  | 0.06 | 0.807 |
| Male | 49 | 34 | 15 |  |  |
| Female | 27 | 18 | 9 |  |  |
| Tumor Size |  |  |  | 3.17 | 0.075 |
| ≤3cm | 46 | 35 | 11 |  |  |
| >3cm | 30 | 17 | 13 |  |  |
| CA19-9 |  |  |  | 0.24 | 0.622 |
| ≤37U/mL | 28 | 19 | 9 |  |  |
| >37U/mL | 48 | 33 | 15 |  |  |
| CEA |  |  |  | 0.90 | 0.344 |
| ≤3.4ng/mL | 32 | 20 | 12 |  |  |
| >3.4ng/mL | 44 | 32 | 12 |  |  |
| Tumor Location |  |  |  | 0.05 | 0.823 |
| Head | 52 | 36 | 16 |  |  |
| Body and Tail | 24 | 16 | 8 |  |  |
| Tumor Differentiation |  |  |  | 0.33 | 0.567 |
| Poor | 22 | 14 | 8 |  |  |
| Moderate and Well | 54 | 38 | 16 |  |  |
| Vascular Invasion |  |  |  | 0.47 | 0.492 |
| No | 62 | 44 | 18 |  |  |
| Yes | 14 | 8 | 6 |  |  |
| Lymph node invasion |  |  |  | 0.81 | 0.368 |
| No | 55 | 36 | 19 |  |  |
| Yes | 21 | 16 | 5 |  |  |

**Table 6 Survival analysis of variable features of PDAC patients**

| **Variable** | **HR** | **95%CI** | **P Value** |
| --- | --- | --- | --- |
| Age (≤60y vs. >60y) | 0.932 | 0.552-1.575 | 0.792 |
| Gender (Male vs. Female) | 1.124 | 0.669-1.888 | 0.066 |
| Tumor Size (≤3cm vs. >3cm) | 1.768 | 0.988-3.164 | 0.055 |
| CA19-9 (≤37U/mL vs. >37U/mL) | 1.170 | 0.703-1.946 | 0.546 |
| CEA (≤3.4ng/mL vs. >3.4ng/mL) | 1.036 | 0.620-1.730 | 0.893 |
| Tumor Location (Head vs. Body and Tail) | 0.594 | 0.341-1.035 | 0.594 |
| Tumor Differentiation (Poor vs. Moderate and Well) | 0.717 | 0.394-1.305 | 0.277 |
| Vascular Invasion (No vs. Yes) | 1.613 | 0.864-3.013 | 0.133 |
| Lymph node invasion (No vs. Yes) | 0.784 | 0.420-1.466 | 0.446 |
| Caprin-1 Level (≤1.6 vs. >1.6) | 1.861 | 1.111-3.117 | 0.018^*^ |

HR: hazard ratio CI: confidence interval
